# Hypoxia-dependent sequestration of an oxygen sensor by a widespread structural motif can shape the hypoxic response - a predictive kinetic model

**DOI:** 10.1186/1752-0509-4-139

**Published:** 2010-10-18

**Authors:** Bernhard Schmierer, Béla Novák, Christopher J Schofield

**Affiliations:** 1 Oxford Centre for Integrative Systems Biology (OCISB), University of Oxford, South Parks Road, Oxford OX1 3QU, UK; 2 Department of Biochemistry, University of Oxford, South Parks Road, Oxford OX1 3QU, UK; 3 Chemistry Research Laboratory, University of Oxford, 12 Mansfield Road, Oxford OX1 3TA, UK

## Abstract

**Background:**

The activity of the heterodimeric transcription factor hypoxia inducible factor (HIF) is regulated by the post-translational, oxygen-dependent hydroxylation of its α-subunit by members of the prolyl hydroxylase domain (PHD or EGLN)-family and by factor inhibiting HIF (FIH). PHD-dependent hydroxylation targets HIFα for rapid proteasomal degradation; FIH-catalysed asparaginyl-hydroxylation of the *C*-terminal transactivation domain (CAD) of HIFα suppresses the CAD-dependent subset of the extensive transcriptional responses induced by HIF. FIH can also hydroxylate ankyrin-repeat domain (ARD) proteins, a large group of proteins which are functionally unrelated but share common structural features. Competition by ARD proteins for FIH is hypothesised to affect FIH activity towards HIFα; however the extent of this competition and its effect on the HIF-dependent hypoxic response are unknown.

**Results:**

To analyse if and in which way the FIH/ARD protein interaction affects HIF-activity, we created a rate equation model. Our model predicts that an oxygen-regulated sequestration of FIH by ARD proteins significantly shapes the input/output characteristics of the HIF system. The FIH/ARD protein interaction is predicted to create an oxygen threshold for HIFα CAD-hydroxylation and to significantly sharpen the signal/response curves, which not only focuses HIFα CAD-hydroxylation into a defined range of oxygen tensions, but also makes the response ultrasensitive to varying oxygen tensions. Our model further suggests that the hydroxylation status of the ARD protein pool can encode the strength and the duration of a hypoxic episode, which may allow cells to memorise these features for a certain time period after reoxygenation.

**Conclusions:**

The FIH/ARD protein interaction has the potential to contribute to oxygen-range finding, can sensitise the response to changes in oxygen levels, and can provide a memory of the strength and the duration of a hypoxic episode. These emergent properties are predicted to significantly shape the characteristics of HIF activity in animal cells. We argue that the FIH/ARD interaction should be taken into account in studies of the effect of pharmacological inhibition of the HIF-hydroxylases and propose that the interaction of a signalling sensor with a large group of proteins might be a general mechanism for the regulation of signalling pathways.

## Background

In animals, the response to hypoxia is mediated by an α,β-heterodimeric transcription factor, the hypoxia inducible factor or HIF. In humans, there are three different HIFα isoforms, with HIF1α and HIF2α being better characterised than HIF3α. The HIFβ subunit is identical with the aryl hydrocarbon receptor nuclear translocator (ARNT). Both the level and transcriptional activity of HIF are regulated by post-translational hydroxylation of the HIFα, but not HIFβ, subunit. In the presence of sufficient oxygen, HIF1α and HIF2α undergo hydroxylation of two proline-residues in their oxygen-dependent degradation domain (ODD), reactions catalysed by three Fe(II)- and 2-oxoglutarate-dependent prolyl hydroxylase domain (PHD1-3 or EGLN1-3) enzymes [1]. In healthy mammalian cells, PHD2 is the most important regulator of the hypoxic response as shown by cellular [2] and animal studies [3]. HIF1α and HIF2α also undergo asparaginyl hydroxylation [4] of the *C-*terminal (CAD) of the two transactivation domains found in HIFα (CAD-hydroxylation). This reaction is catalysed by factor inhibiting HIF (FIH), which is also an Fe(II)- and 2-oxoglutarate-dependent oxygenase [5,6]. HIFα prolyl hydroxylation by PHDs very substantially increases its binding to the von Hippel Lindau protein (pVHL), which acts as a targeting component for an E3 ubiquitin ligase complex and thus mediates rapid degradation of HIFα by the proteasome. When PHD catalysis is limited by oxygen availability, i.e. in hypoxia, HIFα degradation is slowed, its level rises, it dimerises with HIFβ and upregulates HIF-target gene transcription. In contrast to the PHD-dependent ODD-hydroxylation, FIH-dependent CAD-hydroxylation does not affect the stability of HIFα, but more directly decreases the transcriptional activity of HIF by blocking the recruitment of the transcriptional co-regulator p300/CBP to the CAD [4,6], thus disrupting CAD-dependent target gene expression. In contrast, target genes that depend on the *N-*terminal transactivation domain (NAD) of HIFα are not affected by FIH activity [7]. For reviews see [8-10].

More recently, it has become clear that HIFα is not the only FIH substrate, but that FIH also catalyses the hydroxylation of a wide range of other proteins [11-16]. With the notable exception of HIFα itself, all FIH substrates identified to date contain an ankyrin-repeat domain (ARD), an evolutionarily ancient structural domain found in all kingdoms of life [17]. ARDs seem to predominantly mediate protein-protein interactions [18], and occur in proteins as diverse as signal transducers, ion channels, cell cycle regulators, transcriptional regulators and chromatin-associated proteins. ARD proteins contain varying numbers of ankyrin repeats (ARs). ARs are one of the most commonly occurring protein repeats in animals [17]. The stereotypical AR consists of 33 amino acid residues and has an L-shaped fold, which is formed by two short α-helices, arranged in an anti-parallel fashion, and, perpendicular to the helices, a protruding loop region followed by a β-hairpin. The asparagine residue targeted by FIH in some ARs is located in the loop region. ARs stack together to form an ARD, which, in humans, can contain up to 28 ARs. Several studies suggest that AR-hydroxylation by FIH is widespread [11-16], however its biological significance is unclear. Studies with consensus ARDs suggest that hydroxylation may cause an increase in the thermodynamic stability of the ARD fold [19,20], and some evidence points to a potential role for ARD hydroxylation in signalling crosstalk in the cases of Notch [13,16] and NFκB/IκBα [11]. Because the inhibition of HIFα CAD-dependent transcription remains the only well-defined functional outcome of the catalytic activity of FIH, ARD proteins have been speculated to fine-tune FIH activity towards HIFα by binding and sequestering FIH [12,21]. The discovery that FIH interacts with multiple ARD proteins raises major questions as to the role of FIH as an oxygen sensor. To our knowledge, the proposal that the interaction of multiple proteins with a sensor has a regulatory role, is unprecedented. It is unclear what effect the competitive inhibition of FIH by ARD proteins would have on signal processing and on the input/output relation of the network. Because the proposed regulatory effect of ARD proteins on FIH involves multiple interactions it is difficult to study via classical approaches. We therefore devised and analysed a rate equation model of HIFα CAD-hydroxylation.

Our model predicts that the presence of ARD proteins and their hydroxylation by FIH can indeed fine-tune HIFα CAD-hydroxylation, provided that the affinity of FIH for ARD proteins is significantly weakened by their hydroxylation. The simulations highlight unexpected functional consequences of the FIH/ARD protein interaction for the hypoxic response: By creating an oxygen threshold, HIFα CAD-hydroxylation is predicted to be focused into a defined range of oxygen tensions (range finding mechanism), the signal/response curves of HIFα CAD-hydroxylation is predicted to become significantly sharpened (ultrasensitivity), and, upon reoxygenation, FIH-release is predicted to occur with a time-delay, the length of which depends on the duration and the strength of the preceding hypoxic period (memory effect).

## Methods

### A database of human ankyrin repeats (ARs)

The SMART [22], PFAM [23] and Uniprot [24] databases were searched for human AR sequences. SMART contains 1766 human ARs corresponding to 341 distinct protein entries, PFAM Version 24 contains 2337 ankyrin repeats corresponding to 646 protein entries in Uniprot. Sequences that are represented incompletely in the PFAM and SMART databases were extended to the canonical length of 33 residues. To eliminate redundancy, ARs corresponding to different entries for identical proteins were removed. All individual ARs were assembled into a database (Additional File 1), which includes the amino acid sequences of all repeats, the protein names and identifiers, their position of the AR within the ARD protein and a classification according to sequence motifs. ARs longer than 34 or shorter than 32 residues (less than 10% of repeats) were excluded from the set of sequences used to obtain the consensus sequence and the sequence logos.

### Kinetic modelling

#### Nomenclature of kinetic parameters and reaction species

Catalytic rate constants (*k_cat_*) and dissociation constants for enzyme-substrate complexes (*K_D_*) are distinguished by superscripts, the hydroxylation rate functions (*ν*) for the three hydroxylation reactions by subscripts, with *P *for PHD-dependent HIFα ODD-hydroxylation, *FH *for FIH-dependent HIFα CAD-hydroxylation, *FA *and for FIH-dependent ankyrin hydroxylation. $K_{M}^{P}$ and $K_{M}^{F}$ are the Michaelis constants of PHD and FIH for oxygen, respectively. *k_s _*and *k_d _*are the rate constants for basal protein synthesis and degradation, with superscripts *H *and *A *indicating HIFα and ARD proteins, respectively. A list of all model species is given in Table 1, a list of parameters in Table 2.

**Table 1:** List of model species.

Variable species	
*H_tot_*	all HIFα

*H*	non-CAD-hydroxylated HIFα

*H_OH_*	CAD-hydroxylated HIFα

*A*	unhydroxylated FIH target repeats

*A_OH_*	hydroxylated FIH target repeats

**Constant species**	

*A_tot_*	all FIH target ankyrin repeats

*P_tot_*	all PHD

*F_tot_*	all FIH

*O*_2_	intracellular oxygen

**Table 2:** Dimensionless parameter values used for the Full Model.

Number	Parameter description	Parameter	Values	Source
1	PHD concentration	${\hat{P}}_{tot}$	0.2	estimate

2	Dissociation constant of PHD/HIFα binding	${\hat{K}}_{D}^{P}$	1	[29]

3	(*k_cat _*for ODD-hydroxylation)/(basal HIFα degradation rate constant)	${k^{\prime }}_{cat}^{P}$	500	[31]

4	FIH concentration	${\hat{F}}_{tot}$	1	estimate

5	Dissociation constant of FIH/HIFα binding	${\hat{K}}_{D}^{FH}$	1	set to same as 2

6	(*k_cat _*for HIFα CAD-hydroxylation)/(*k_cat _*for HIFα ODD-hydroxylation)	$\omega =\frac{K_{cat}^{FH}}{K_{cat}^{FA}}$	1	estimate

7	(K_M _of FIH for oxygen)/(K_M _of PHD for oxygen)	$\alpha =\frac{K_{M}^{F}}{K_{M}^{P}}$	0.33	[30]

8	total concentration of FIH target ARs	${\hat{A}}_{tot}$	0 - 500	varied

9	Dissociation constant of FIH/ARD protein binding (unhydroxylated ARs)	${\hat{K}}_{D}^{FA}$	1	set to same as 2

10	(FIH affinity for hydroxylated)/(FIH affinity for unhydroxylated ARs)	*γ*	0 - 0.1	varied

11	(basal HIFα degradation rate constant)/(AR degradation rate constant)	$\varepsilon =\frac{k_{d}^{H}}{k_{d}^{A}}$	1 - 10	varied

12	Dissociation constant of HIF/HRE binding	${\hat{K}}_{D}^{HRE}$	0.3	estimate

13	Oxygen concentration	${\tilde{O}}_{2}$	0 - 1	varied

#### Modelling and modelling assumptions

For the HIF-hydroxylases to act as oxygen sensors in the proposed manner, their activity in cells must be limited by oxygen availability. Moreover, to ensure that HIFα-levels and the amount of HIF bound to DNA reflect the intracellular oxygen tension at all times, ODD-hydroxylation must be rate-limiting rather than degradation of ODD-hydroxylated HIFα, complex formation with HIFβ or DNA-binding of HIF. These requirements allow us to make the following simplifying assumptions: Firstly, HIFβ and the hydroxylase co-substrates, 2-oxoglutarate and Fe(II), are not limiting, and secondly, the degradation of ODD-hydroxylated HIFα as well as binding of HIF to hypoxia response elements are fast compared to the ODD-hydroxylation reaction. Although we appreciate that under some conditions these assumptions may not be valid, for instance in some tumour cells [25,26], a body of evidence suggests that these assumptions are reasonable for normal cells. All simulations were done using the open source software XPP-AUT [27]. Steady state values were calculated by running time course simulations at different oxygen-tensions until a steady state was reached.

#### The Full Model

For the hydroxylation rate functions in the Full Model, we take into account that free HIFα concentration decreases by binding to the enzymes ("full model kinetics", see also Additional File 2). Unlike classical Michaelis-Menten kinetics, this approach is also valid if there is no substrate excess, a situation that is frequently encountered in protein-protein interaction networks [28]. Because of the expected excess of ARD proteins over FIH, we use the Michaelis-Menten approximation for FIH-catalysed AR-hydroxylation. The Full Model is defined by three ordinary differential equations, which are given in dimensionless form. The CAD-hydroxylated forms of HIFα and the ARD proteins are defined by mass conservation. "Hat" (^) indicates non-dimensional quantities expressed relative to the maximal amount of HIFα present in the absence of oxygen, and "prime00000000000" (') indicates non-dimensional quantities expressed relative to the basal degradation rate constant for HIFα. Oxygen is given relative to the K_M _of PHD for oxygen, which is indicated by "tilde" (~). For details, refer to Additional File 2.

(1) $$\frac{d{\hat{H}}_{tot}}{d\tau }=1-{\hat{H}}_{tot}(1+{\overset{´}{\nu }}_{P})$$

(2) $$\frac{d\hat{H}}{d\tau }=1-\hat{H}(1+{\overset{´}{\nu }}_{P}+{\overset{´}{\nu }}_{FH})$$

(3) $$\varepsilon \frac{d\hat{A}}{d\tau }={\hat{A}}_{tot}-\hat{A}(1+\varepsilon {\overset{´}{\nu }}_{FA})$$

The hydroxylation rate functions *ν_P_*, *ν_FH _*and *ν_FA _*(Additional File 2, Sections 2-4) are given in dimensionless form (Additional File 2, Section 5) by the following expressions:

1. PHD-dependent HIFα ODD-hydroxylation(${\overset{´}{\nu }}_{P}$)

(4) $${\overset{´}{\nu }}_{P}={\overset{´}{k}}_{cat}^{P}{\hat{P}}_{tot}\frac{1}{{\hat{K}}_{D}^{P}+{\hat{H}}_{P}+{\hat{P}}_{tot}}\left(\frac{{\tilde{O}}_{2}}{1+{\tilde{O}}_{2}}\right)$$

(5) $${\hat{H}}_{P}=\frac{1}{2}\left({\hat{H}}_{tot}-{\hat{P}}_{tot}-{\hat{K}}_{D}^{P}+\sqrt{{\left({\hat{K}}_{D}^{P}+{\hat{P}}_{tot}-{\hat{H}}_{tot}\right)}^{2}+4{\hat{K}}_{D}^{P}{\hat{H}}_{tot}}\right)$$

${\hat{H}}_{P}$ indicates HIFα that is not already bound to PHD, i.e. the fraction of HIFα free to bind PHD, whether it is CAD-hydroxylated or not.

2. FIH-dependent HIFα CAD-hydroxylation in the presence of competing ARs (${\overset{´}{\nu }}_{FH}$)

(6) $${\overset{´}{\nu }}_{FH}={\overset{´}{k}}_{cat}^{FH}{\hat{F}}_{tot}\frac{1}{{\hat{K}}_{i}^{FH}+{\hat{H}}_{F}+{\hat{F}}_{tot}}\left(\frac{{\tilde{O}}_{2}}{\alpha +{\tilde{O}}_{2}}\right)$$

(7) $${\hat{H}}_{F}=\frac{1}{2}\left(\hat{H}-{\hat{F}}_{tot}-{\hat{K}}_{i}^{FH}+\sqrt{{\left({\hat{K}}_{i}^{FH}+{\hat{F}}_{tot}-\hat{H}\right)}^{2}+4{\hat{K}}_{i}^{FH}\hat{H}}\right)$$

(8) $${\hat{K}}_{i}^{FH}\overset{\text{def}}{=}{\hat{K}}_{D}^{FH}\left(1+\frac{\hat{A}}{{\hat{K}}_{D}^{FA}}+\frac{\gamma {\hat{A}}_{OH}}{{\hat{K}}_{D}^{FA}}\right)$$

${\hat{H}}_{F}$ indicates non-CAD-hydroxylated HIFα not already bound to FIH, i.e. the fraction of HIFα free to bind FIH. For a definition of *α *and *γ *see Table 2.

3. FIH-dependent AR-hydroxylation in the presence of competing HIFα(${\overset{´}{\nu }}_{\text{FA}}$), where ${\hat{H}}_{\text{F}}$ is given by Eq. 7:

(9) $${\overset{´}{\nu }}_{FA}={\overset{´}{k}}_{cat}^{FA}{\hat{F}}_{tot}\frac{1}{{\hat{K}}_{D}^{FA}\left(1+\frac{{\hat{H}}_{F}}{{\hat{K}}_{D}^{FH}}\right)+\hat{A}+\gamma {\hat{A}}_{OH}}\left(\frac{{\tilde{O}}_{2}}{\alpha +{\tilde{O}}_{2}}\right)$$

#### Skeleton Model 1 (SKM1)

SKM1 ignores the presence of ARD proteins and approximates both HIFα hydroxylation rate functions (${\overset{´}{\nu }}_{P},{\overset{´}{\nu }}_{FH}$) by Michaelis-Menten kinetics. We are left with the differential equations Eq. 1 and Eq. 2 only. The hydroxylation rate functions, Eq. 4 and Eq. 6, simplify to

(10) $${\overset{´}{\nu }}_{P}={\overset{´}{k}}_{cat}^{P}{\hat{P}}_{tot}\frac{1}{{\hat{K}}_{D}^{P}+{\hat{H}}_{tot}}\left(\frac{{\tilde{O}}_{2}}{1+{\tilde{O}}_{2}}\right)$$

(11) $${\overset{´}{\nu }}_{FH}={\overset{´}{k}}_{cat}^{FH}{\hat{F}}_{tot}\frac{1}{{\hat{K}}_{D}^{FH}+\hat{H}}\left(\frac{{\tilde{O}}_{2}}{\alpha +{\tilde{O}}_{2}}\right)$$

If binding of HIF to the DNA is fast, the fractions of hypoxia-response elements (HRE) occupied with a non-CAD-hydroxylated or CAD-hydroxylated HIFα, respectively, are described by the steady state expressions

$$HRE_{CAD}=\frac{\hat{H}}{{\hat{K}}_{D}^{HRE}+{\hat{H}}_{tot}}\;\;HRE_{CADOH}=\frac{{\hat{H}}_{OH}}{{\hat{K}}_{D}^{HRE}+{\hat{H}}_{tot}}$$

the sum of which is the fraction of HREs occupied by either form. Here, $K_{D}^{HRE}$ is the dissociation constant of the HIF/HRE interaction, assuming all forms of HIF bind to DNA with the same affinity. These expressions can also be interpreted as the probability of a specific HRE to be occupied by either species.

#### Skeleton Model 2 (SKM2)

SKM2 ignores HIFα and describes FIH/ARD protein interactions only, using the differential equation Eq. 12. The fraction of FIH that is not bound to ARD proteins is given by Eq. 13. In contrast to the Full Model and SKM1, in SKM2, "hat" (^) indicates non-dimensional quantities expressed relative to the total amount of FIH-target ankyrin repeats. In SKM2, oxygen is given relative to the *K_M _*of FIH for oxygen, which is indicated by "dash" (-). The parameters *κ *"sequestration capacity") and *β *("hydroxylation efficiency") are defined in Eq. 14 and 15. See also Additional File 2, Section 6.

(12) $$\frac{d\hat{A}}{d\sigma }=1-\hat{A}-\beta \frac{\kappa \hat{A}}{1+\kappa \hat{A}}\frac{{\bar{O}}_{2}}{1+{\bar{O}}_{2}}$$

(13) $$F_{free}=\frac{1}{1+\kappa \hat{A}}$$

(14) $$\kappa \overset{\text{def}}{=}\frac{A_{tot}}{K_{D}^{FA}}$$

(15) $$\beta \overset{\text{def}}{=}\frac{k_{cat}^{FA}F_{tot}}{k_{d}^{A}A_{tot}}$$

### Parameter values

Experimental information restricts the biologically relevant range of parameter values and allows estimates. The K_M _of PHDs for oxygen has been reported to be in the range of approximately 220-250 μM [29] and references therein. This is slightly higher than the maximal solubility of oxygen in water, which sets a theoretical upper limit for intracellular oxygen concentrations. Thus, the biologically relevant range of oxygen-tensions must be below this value. The K_M _of FIH for oxygen has been reported to be lower than the K_M _of the PHDs [30], which we take into account. We normalise to the maximal amount of HIFα, $H_{tot}^{\max}=1$, which is reached at steady state in the absence of oxygen-dependent degradation. Three parameters define HIFα ODD-hydroxylation, the dissociation constant of the PHD/HIFα interaction, ${\hat{K}}_{D}^{P}$; the maximal reaction rate, $\nu_{max}^{P}$; and the PHD concentration, *P_tot_*. We used the reported value of 1 μM for the PHD/HIFα binding affinity [29], i.e. ${\hat{K}}_{D}^{P}=1$. We then chose $\nu_{max}^{P}$ such that a good agreement with measured signal/response curves was obtained [31]. PHD expression levels relative to HIFα are not known, and the concentration of PHD was set to 0.2, an assumption which does not affect any of our conclusions. In our simulations, the overall rate of FIH hydroxylation must be higher than the overall rate of PHD hydroxylation for significant HIFα CAD-hydroxylation to occur. The means by which high FIH activity is achieved are irrelevant for our conclusions (data not shown), whether by higher expression levels, higher affinity for HIFα or a faster turnover rate. We thus introduce an arbitrary five-fold excess of FIH over PHD, which allows us to clearly illustrate the inhibitory effect of the ARD proteins on FIH activity. The binding affinity of FIH for HIFα and the turnover rate are set to match the values for PHD. *In vivo*, FIH activity might be lower than assumed in the model, however all our conclusions are qualitative and thus entirely independent of these assumptions. Table 2 summarises the dimensionless parameter values that were used for calculating the graphs shown, unless indicated otherwise in the figure legends. The model can be found in the BioModels Database, http://www.ebi.ac.uk/biomodels/ accession number MODEL1008170000.

## Results

### Potential ankyrin-type FIH targets in the human proteome

To investigate the potential extent of ARD protein interaction with FIH, we initially carried out bioinformatic analyses. Searching the SMART [22], PFAM [23] and Uniprot [24] databases for human AR-sequences, we found 1505 annotated ankyrin repeats (ARs) mapping to 252 distinct human ARD proteins. All 1505 ARs were assembled into a database (Additional File 1) and analysed for the presence of a potential FIH hydroxylation site. Diagnostic features were then extracted from experimentally verified target sequences to aid the prediction of AR-type FIH substrates and give an estimate of their overall abundance.

**Figure 1:**
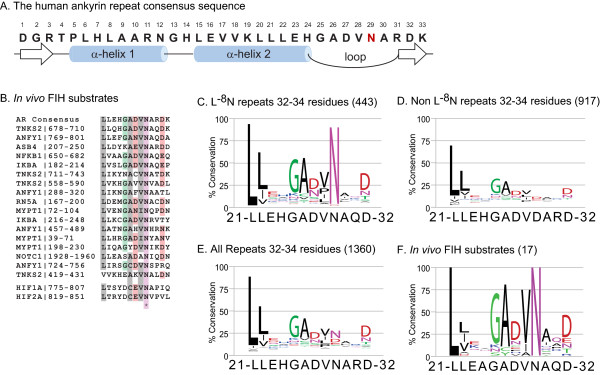
**Bioinformatic analysis of human ankyrin repeats**. A. The human ankyrin repeat consensus sequence. Asn-29 in the loop region is the hydroxylation site. The consensus sequence obtained differs at position 1 (N -> D) and position 31 (K -> R) from a previously reported consensus obtained by aligning ARs without restriction to a particular species [41]. Secondary structural elements are indicated. B. Experimentally verified *in vivo *hydroxylation targets of FIH. Conserved residues are highlighted. The ankyrin consensus as well as the hydroxylation regions of HIF1/2α are given for comparison. C-F. Sequence alignments. Different subgroups of human ankyrin repeats were aligned and displayed as sequence logos [32]. The analysis was restricted to repeats with a length of 32-34 residues (~90% of repeats). Note the much higher degree of conservation in L^-8^N repeats (C) when compared to non-L^-8^N repeats (D). Alignments of all human ARs (E), and of verified ankyrin-type *in vivo *substrates of FIH (F) are given for completion.

The minimal requirement for a specific AR to be an FIH substrate is the presence of a hydroxylatable residue in the loop region downstream of the second α-helix, located at position 29 of the prototypical 33 residue ankyrin repeat consensus sequence (Figure 1A). A total of 922 human AR (61%) were found to contain one or more asparagines within the loop region, i.e. between positions 25 and 33 of the repeat. This subgroup was termed Asn-repeats, and all are potential FIH targets. The remaining 584 human AR (39%) lack an asparagine between consensus positions 25 and 33 (non-Asn-repeats). Experimental studies have identified a number of ARs that are hydroxylated by FIH either *in vivo *or at least *in vitro *(Additional File 3, Table S1), or interact with FIH (Additional File 3, Table S2). Comparison of the sequences of ARs that are hydroxylated *in vivo *with the hydroxylation motif in the *C*-terminal transactivation domain of HIFα reveals several conserved features that might allow prediction of whether or not a given AR is an FIH target (Figure 1B). All but one FIH target-repeats verified as such *in vivo *have a leucine residue at position -8 relative to the hydroxylated asparagine (L^-8^N group), which is also present in HIFα and in the AR consensus sequence. Other features found in HIFα that are also commonly present in verified ankyrin-type *in vivo *substrates of FIH are a hydrophobic residue (valine or isoleucine) at position -1 relative to Asn-29, a polar residue such as glutamic acid or aspartic acid/asparagine in position -2, and an alanine or cysteine at position -3 relative to the Asn-29, which may be important in binding to the active site of FIH [13]. To investigate a possible correlation between the presence of an L^-8^N motif and the conservation of the residues at positions -3, -2 and -1 relative to Asn-29, we compared alignments of L^-8^N repeats (Figure 1C) and ARs lacking an L^-8^N motif (Figure 1D). Sequence logos [32] of alignments of all ARs (Figure 1E) as well as of experimentally verified hydroxylation targets (Figure 1F) are shown for comparison. We found that the features conserved between HIFα and verified ankyrin-type hydroxylation targets are enriched in the subgroup of L^-8^N-repeats compared to ARs without an L^-8^N motif (compare Figures 1C and 1D). Thus, the presence of an L^-8^N motif seems to correlate with the presence of several additional features that are also present in HIFα, and thus appears to define a subgroup of human ARs, which likely contains most FIH target repeats. It is tempting to speculate that the higher degree of conservation found in positions -1, -2 and -3 relative to Asn-29 has been maintained in the L^-8^N group because these residues are important for FIH-dependent hydroxylation. However, other explanations such as structural requirements are also possible.

Overall, we analysed and classified 1505 individual human ankyrin repeats (Table 3). We find that 472 human ankyrin repeats (31%) in 182 distinct ARD proteins contain an L^-8^N motif. Applying more stringent criteria, the most likely ankyrin-type hydroxylation targets of FIH have the consensus sequence *21-L(X)_4_(AC)(DEN)(ILV)N-29*, which is found in 166 ARs mapping to 105 ARD proteins. Thus, 166 likely is a lower limit for the number of ankyrin-type FIH asparaginyl-hydroxylation targets. The large number of AR-type FIH targets supports the idea that FIH-hydroxylatable ARD proteins can compete with HIFα for FIH binding. How this would contribute to the suggested "fine-tuning" of HIF-regulation is however not obvious, because a strong, constitutive sequestration of FIH by ARD proteins would merely attenuate FIH activity towards HIFα at all oxygen concentrations. Also, the affinity of FIH for ARDs can be higher than its affinity for HIFα [33,34], and it is difficult to envisage how HIFα CAD-hydroxylation can occur at all in the presence of such a large pool of efficient competitors. However, hydroxylation of ARs has been shown to significantly decrease their affinity for FIH [13], suggesting that FIH, by hydroxylating ARs in an oxygen dependent manner, is able to trigger its own release from ARD protein sequestration [12,14,21]. To test this proposal and to investigate its implications for the hypoxic response, we devised and analysed a rate equation model describing these processes.

**Table 3:** Classification of ankyrin-repeats according to sequence motifs.

Group	Total	% of all AR	Asn-hydroxylation targets?
all L^-8^N,	472	31%	Likely

of which L(X)_4_(AC)(DEN)(ILV)N	166	11%	

Asn-repeats, non L^-8^N	449	30%	Unlikely

Non-Asn-repeats	584	39%	No

**Total**	**1505**	**100%**	

### Kinetic modelling

**Figure 2:**
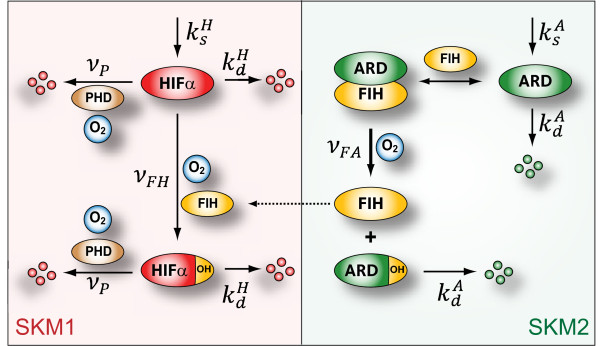
**Schematic of the reaction network**. Model reactions are indicated by bold arrows, the corresponding rate constants (*k*) or rate functions (*ν*) are indicated. Note that here complex formation between FIH and ARD proteins is shown explicitly for clarity, however in the model this reaction is assumed at steady state and implicit in the AR-hydroxylation rate function, *ν_FA_*. Also, binding of FIH to hydroxylated ARs is accounted for in the Full Model, but not shown in this schematic. Skeleton Models 1 (SKM1, red shading) and 2 (SKM2, green shading) describe subsystems of the full system. ARD, ankyrin-repeat domain proteins. $k_{s}^{A}$, $k_{s}^{H}$, synthesis rates of ARDs and HIFα, respectively. $k_{d}^{A}$, $k_{d}^{H}$, basal degradation rates of ARDs and HIFα, respectively. *ν_P_*, *ν_FH _*and *ν_FA_*, rate functions for HIFα ODD-, HIFα CAD-and ARD protein-hydroxylation, respectively.

We set out to model the scenario that, under normoxic conditions, when the catalytic activity of FIH is high, more ARs will be hydroxylated and thus less able to bind and sequester FIH, a larger proportion of which will be free to target HIFα. When oxygen becomes limiting under hypoxic conditions, FIH catalytic activity will decrease, the fraction of unhydroxylated ARs will rise, and FIH will be sequestered from HIFα more efficiently. In a modular approach, we first analysed two skeleton models, each aimed at capturing the essence of a distinct aspect of the system (Figure 2). Skeleton Model 1 (SKM1) describes the reactions affecting HIFα (basal HIFα turnover, ODD-hydroxylation-induced degradation of HIFα, HIFα CAD-hydroxylation), but ignores the presence of ARD proteins. Skeleton Model 2 (SKM2) describes the reactions affecting ARD proteins (basal ARD protein turnover, FIH binding to ARD proteins, their hydroxylation, and hydroxylation-dependent FIH-release). Because of their parsimony, the skeleton models provide significant insight into the logic of these processes. Both skeleton models were then fused into a Full Model, which we used to study if and how oxygen-regulated competitive inhibition by ARD proteins can shape the hypoxic response. The XPP-AUT modelling file is provided as Additional File 4.

### Skeleton Model 1 - HIFα CAD-hydroxylation in the absence of the FIH/AR-interaction

**Figure 3:**
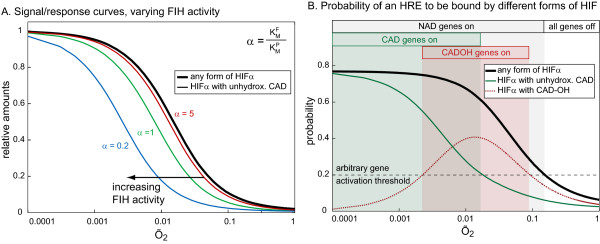
**Signal/response curves in Skeleton Model 1**. A. Increasing FIH activity shifts non-CAD-hydroxylated HIFα to lower oxygen tensions. Total HIFα (black bold line) and HIFα that is not CAD-hydroxylated (coloured fine lines) are shown as functions of oxygen. Parameters used were ${\hat{K}}_{D}^{P}={\hat{K}}_{D}^{F}=1,{\overset{´}{k}}_{cat}^{P}{\hat{P}}_{tot}={\overset{´}{k}}_{cat}^{FH}{\hat{F}}_{tot}=\;100$. In this example, FIH activity was increased by lowering its K_M _for oxygen relative to PHD's K_M _for oxygen, however any change in parameters that leads to higher FIH activity gives similar results (data not shown). The green curve (*α *= 1) represents the case where all kinetic parameters for FIH and PHD are identical. B. Activation status of hypoxia-response elements (HREs). Probabilities for an HRE to be bound by either HIFα that is not CAD-hydroxylated (green solid curve), by CAD-hydroxylated HIFα (red dotted curve) or either form of HIFα (bold black curve) under the assumption that all forms of HIF bind with identical affinity to the HRE(${\hat{K}}_{D}^{HRE}=0.3$). We propose the existence of a novel set of HIF target genes, which are transcriptionally activated by CAD-hydroxylated HIFα (CADOH-dependent genes). The regimes of differential gene expression are indicated by shading (an arbitrary gene activation threshold was introduced at 20% HRE occupancy). Parameters used were as in A, with *α *= 0.33[30].

By targeting HIFα for degradation in an oxygen-dependent manner, PHD activity restricts the amount of HIFα that is available for FIH-dependent HIFα CAD-hydroxylation (Figure 3A, bold black curve). Oxygen-dependent FIH activity then determines the fraction of total HIFα that is CAD-hydroxylated. Increasing FIH activity shifts the curve of non-CAD-hydroxylated HIFα to the left towards lower oxygen-tensions (Figure 3A). Thus, the higher FIH activity, the more severe the hypoxia that is required to induce transcription of CAD-dependent target genes. The model predicts two regions of distinct gene expression, which are defined by the oxygen-tension (Figure 3B). A decrease in oxygen-levels first leads into a regime where hypoxia response elements (HRE) start to become occupied by accumulating HIF, however a lot of the occupying HIF contains CAD-hydroxylated HIFα. In this region, NAD-dependent genes are expressed (Figure 3B, grey shading), but CAD-dependent genes remain repressed. The model predicts that a further decrease in oxygen-levels will cause non-CAD-hydroxylated HIFα to increase sufficiently to allow expression of CAD-dependent genes. In this regime, both NAD- and CAD-dependent genes are expressed (Figure 3B, green shading). The prediction that more severe hypoxia is required to activate CAD-dependent genes than to activate NAD-dependent genes is consistent with previous experimental results [7].

Experimentally, CAD-dependent genes, but not NAD-dependent genes, are induced by FIH knockdown and repressed by FIH overexpression [7]. Interestingly, a third group of HIF target genes have been described, which show the opposite behaviour to CAD-dependent genes. This group, one member of which is *BNIP3 *(BCL2/adenovirus E1B 19 kD interacting protein 3), is repressed by FIH knockdown and de-repressed by FIH overexpression [7,35]. To explain this unexpected behaviour, a CAD-dependently expressed repressor was postulated, which would be present only at very low oxygen concentrations [7]. Our model suggests a more parsimonious explanation, which can explain the unusual behaviour of some HIF target genes. We propose that *BNIP3 *belongs to a third, novel class of target genes, which are specifically activated by the CAD-hydroxylated HIFα (CADOH-dependent, Figure 3B, red shading), and thus expressed in a p300/CBP-independent manner. This proposal assigns a potential direct function to CAD-hydroxylated HIFα, and we studied the behaviour of this species in more detail. Because FIH activity is a monotonically increasing function of oxygen, the naive expectation is that the amount of CAD-hydroxylated HIFα should also increase monotonically (at least until a saturation point) with increasing oxygen-levels. The model predicts however that this is not the case and that the level of CAD-hydroxylated HIFα will peak at intermediate oxygen tensions. The location and magnitude of the peak of CAD-hydroxylated HIFα are parameter dependent, but its existence is a generic system property. The postulated group of CADOH-dependent genes is thus predicted to be expressed at intermediate hypoxia only (Figure 3B), and to show the bell-shaped signal/response curve observed experimentally [7].

### Skeleton Model 2 - FIH sequestration by ARD proteins and oxygen-dependent FIH-release

**Figure 4:**
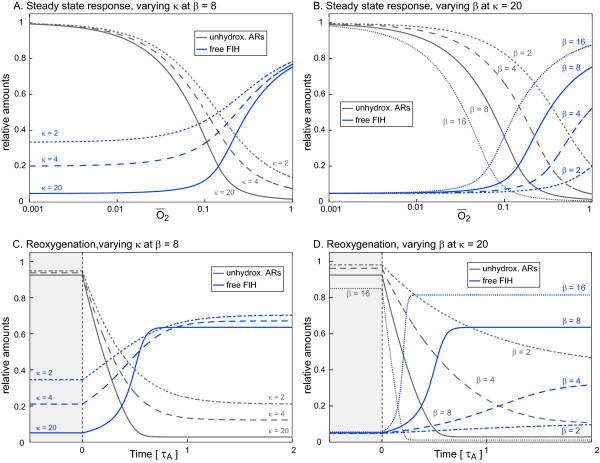
**Skeleton Model 2. FIH-release is determined by the FIH sequestration capacity of the ARD protein pool, and by the efficiency of AR-hydroxylation**. The sequestration capacity *κ *depends on the total concentration of ARs and on their affinity for FIH. The hydroxylation efficiency *β *relates the maximal rate of AR hydroxylation to the average turnover rate of ARD proteins. Free FIH (blue curves) and unhydroxylated, FIH sequestering ankyrin repeats (grey curves) are plotted either against ${\bar{O}}_{2}$, the oxygen concentration relative to the K_M _of FIH for oxygen (A and B) or against time expressed in units of the mean life time of an average ARD protein (C and D). A. Signal/response curves. Varying *κ* strongly affects the dynamic range of free FIH. If *κ *is larger, FIH is sequestered more efficiently in the absence of oxygen. FIH-release is sharper and the dynamic range of free FIH is increased. Note that varying *κ *does not significantly affect the oxygen-threshold of FIH-release from AR-dependent sequestration. B. Signal/response curves. *β *sets the oxygen-threshold for FIH-release. Varying *β *strongly affects the oxygen-tension at which FIH-release occurs, however does not affect the slope of the signal/response curves. C and D. Time-resolved response to reoxygenation after hypoxia. The system was equilibrated at ${\bar{O}}_{2}$ = 0.01 (hypoxia, light grey area). At *t *= 0, oxygen was increased to ${\bar{O}}_{2}$ = 0.5 (reoxygenation) and the system response was simulated. C. Varying *κ *changes the sharpness, but not the timing of the response. Increasing *κ *sharpens the time-dependence of FIH-release. Note the time delay until half-maximal response (about 0.5 time units), which is insensitive to variations in *κ*. D. Varying *β *changes both the sharpness and the timing of the response. The larger *β*, the more rapid and more pronounced is the FIH-release.

By ignoring the presence of HIFα, SKM2 reduces the system to the sequestration of FIH by ARD proteins, their hydroxylation, and the subsequent oxygen-dependent FIH-release and allows us to define the requirements for an "efficient", FIH-release, which we define as a clear differential between the levels of free FIH at high and low oxygen concentrations. Two non-dimensional parameters determine the system behaviour (Figure 4): The sequestration capacity of the ARD protein pool (*κ*), which depends on the total concentration of ARs and their affinity for FIH; and the hydroxylation efficiency (*β*), which is the *ν_max _*of the hydroxylation rate of ARD proteins relative to their degradation rate, and thus relates the rate of AR-hydroxylation to the rate of ARD protein turnover. The signal/response curves (Figures 4A, B) indicate that an efficient FIH-release requires both the sequestration capacity *κ *and the hydroxylation efficiency *β *to be large. At low oxygen-tensions, the steady state is largely determined by *κ*, and the larger *κ*, the lower the level of free FIH (Figure 4A). At high oxygen-tensions, the steady state is determined chiefly by *β*. A large *β *value causes a large fraction of ARs to be in the hydroxylated state and thus incapable of retaining FIH, particularly at high oxygen-levels (Figure 4B). The conditions that allow an efficient and sharp oxygen-dependent release of FIH from ARD protein-dependent sequestration are identical to those that favour near-complete hydroxylation of FIH-target repeats. Thus, SKM2 predicts that near-complete hydroxylation of the FIH-accessible fraction of hydroxylatable target repeats is required for efficient, oxygen-dependent FIH-release.

In addition to this steady state analysis, we also simulated the temporal response of the system to step changes in oxygen concentrations, i.e. to sudden hypoxia and to sudden reoxygenation after a hypoxic episode. SKM2 predicts that the time-resolved response to hypoxia is rather insensitive to variations of *κ *and *β*, with the family of curves showing a hyperbolic decrease in free FIH with time, either reaching distinct steady state values at low oxygen when *κ *is varied (Additional File 3, Figure S1A), or starting out from distinct steady state values at high oxygen if *β *is varied (Additional File 3, Figure S1B). More interestingly however, the temporal response to an increase of oxygen after hypoxia (reoxygenation) is affected significantly by both, *κ *and *β*. Although variations in *κ *affect half-response times for FIH-release only moderately (Figure 4C), varying *κ *does significantly mould the shape of the time-response curves, which change from a gradual FIH-release if *κ *is small, to a delayed, switch-like FIH-release if *κ *is large (Figure 4C). In contrast to varying *κ*, varying *β *substantially affects half response times - the larger *β*, the faster the FIH release in response to reoxygenation (Figure 4D). Thus, SKM2 predicts that oxygen-dependent FIH-release from ARD proteins upon sudden reoxygenation of hypoxic cells can occur in a switch-like manner, with a time delay relative to the reoxygenation event. The reason for this time delay is that, shortly after reoxygenation, the concentration of unhydroxylated, FIH-accessible ARs is still high enough to allow rebinding of any released FIH. SKM2 thus predicts that a permanent release of FIH is only achieved once the FIH-accessible ARs are hydroxylated to a significant extent, and the concentration of unhydroxylated ARs starts to become limiting for FIH sequestration, which, due to the excess of ARs over FIH, can only happen if hydroxylation of FIH-accessible ARs approaches completion. The time required for FIH to hydroxylate ARs to a sufficient degree to overcome sequestration explains the time delay between reoxygenation and FIH-release. This behaviour also allows modulation of the timing of FIH-release as a function of the strength and duration of the preceding hypoxic episode, a feature we explore using the Full Model at the end of the following section.

### The Full Model - the effects of the FIH/ARD protein interaction on HIFα CAD-hydroxylation

**Figure 5:**
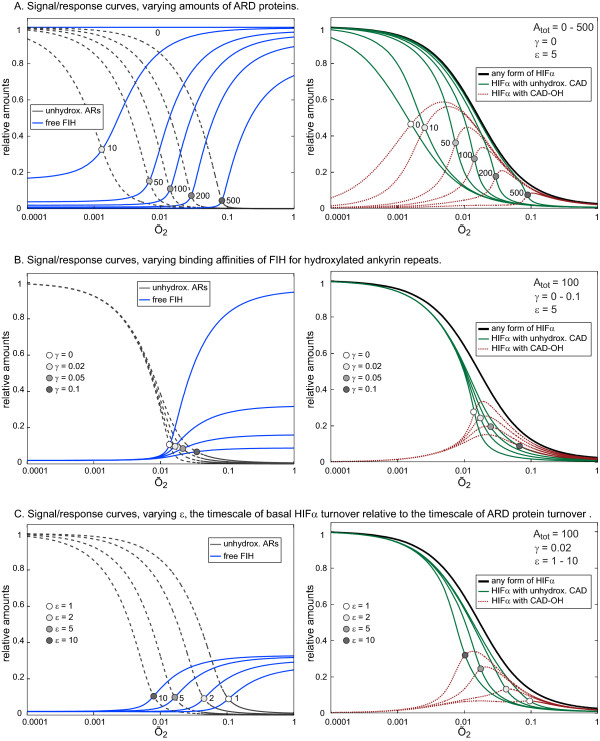
**Full Model, signal/response curves for varying parameter values**. Steady state concentrations of free FIH (blue), and of unhydroxylated FIH-sequestering ARs (grey, left hand panels), as well as of total HIFα (black), non-CAD-hydroxylated HIFα (green) and CAD-hydroxylated HIFα (red, right hand panels) are shown as functions of oxygen. Corresponding pairs of curves are marked at their intersection. A. The amount of ARs (*A_tot _*) determines the oxygen threshold for FIH-release and HIFα CAD-hydroxylation. The larger the number of ARs, the higher the oxygen-tension at which FIH is released (left hand panel). Competing ARs also create an oxygen-threshold for CAD-hydroxylation, above which non-CAD-hydroxylated HIFα drops much more sharply with increasing oxygen levels than in the case where there is no competition (right hand panel, green solid curves). In addition, the FIH/AR-interaction restricts the presence of CAD-hydroxylated HIFα to moderate hypoxia and decreases its peak values (right hand panel, red dotted curves). In contrast to B and C, here FIH is assumed to not bind to hydroxylated ARs at all. B. Binding of FIH to hydroxylated ARD proteins has to be weak for efficient FIH-release. *γ*, the FIH affinity for hydroxylated relative to unhydroxylated ARs, must be small for both efficient FIH-release (left hand panel) and efficient HIFα CAD-hydroxylation (right hand panel). C. ARD proteins must be stable compared to the basal stability of HIFα for efficient HIFα CAD-hydroxylation to occur. Large *ε *indicates stable ARD proteins, which allows FIH-release to occur at lower oxygen levels (left hand panel). Large *ε *increases extent and sharpness of HIFα CAD-hydroxylation and shifts the peak value to more severe hypoxia (right hand panel).

Having simulated the interdependency of HIFα CAD-hydroxylation and oxygen-dependent degradation of HIFα (SKM1), as well as the requirements for efficient, oxygen-dependent release of FIH from ARD proteins (SKM2), we next fused the skeleton models into a Full Model, which we use to investigate the effects of ARD protein hydroxylation on the steady state levels and the temporal response of HIFα CAD-hydroxylation. If the conditions for efficient FIH-release as previously defined by analysis of SKM2 are met, the Full Model predicts a similar sharp and efficient FIH-release above an oxygen threshold, and additionally suggests that this feature is robustly retained over a wide range of ARD protein concentrations. The position of the oxygen threshold for FIH-release is predicted to be determined by the total number of ARs that are FIH targets (Figure 5A, left hand panel). Importantly, the oxygen-threshold for FIH-release translates into an oxygen-threshold for HIFα CAD-hydroxylation (Figure 5A, right hand panel). Below the threshold, non-CAD-hydroxylated HIFα is kept at high levels. The drop at the oxygen threshold is much sharper than in the absence of an FIH/AR-interaction (green solid curves, compare *A_tot _*= 0 with e.g. *A_tot _*= 100, see also Figure S2). Increasing the number of FIH-accessible ARs shifts the oxygen threshold and the decreasing peak values of CAD-hydroxylated HIFα towards higher oxygen-tensions and narrows the oxygen range in which CAD-hydroxylated HIFα can accumulate (Figure 5A, right hand panel). Overall, the Full Model predicts that competition by ARD proteins for FIH introduces an ultrasensitive dependence of HIFα CAD-hydroxylation on oxygen levels. The position of the oxygen threshold above which HIFα CAD-hydroxylation occurs is determined by the concentration of AR-type hydroxylation targets, as is the range of oxygen tensions into which HIFα CAD-hydroxylation is focused. We conclude that the FIH/AR-interaction can cause HIFα CAD-hydroxylation to respond ultrasensitively to changing oxygen levels, and that it can contribute to determining the oxygen-range or the degree of hypoxia at which CAD-hydroxylated HIFα will accumulate.

Up to now we have assumed that FIH does not bind at all to hydroxylated ARs. To make the Full Model more realistic, we now allow such product binding to happen and introduce a parameter 0 <*γ *< 1, which is the binding affinity of FIH for hydroxylated ARs relative to its affinity for unhydroxylated ARs. It becomes clear that even weak binding of FIH to hydroxylated ARs (*γ *< 1) can strongly attenuate the levels of free FIH at high oxygen tensions (Figure 5B, left hand panel). The peak values of CAD-hydroxylated HIFα (Figure 5B, right hand panel) are also decreased. Importantly however, while binding of FIH to hydroxylated ARs attenuates the steepness of the response curve, the existence of an oxygen threshold is entirely independent of such binding. Experiments suggest that there is indeed a substantial differential between the FIH binding affinity for non-CAD-hydroxylated versus CAD-hydroxylated ARs [13], and we predict that this difference is essential for achieving significant HIFα CAD-hydroxylation and the sharpest possible response curves.

FIH-dependent Asn-hydroxylation is, to the best of our knowledge, irreversible and can only be reversed indirectly by degradation of the Asn-hydroxylated proteins and *de novo *synthesis of unhydroxylated proteins. Thus, the mean life times of the competing substrates, ARD-proteins and HIFα, are expected to be important parameters. To test this, we varied the parameter *ε*, which is the ratio of the mean life time of ARD-proteins relative to the mean life time of HIFα under basal turnover conditions, i.e. in the absence of oxygen. The Full Model predicts that long-lived ARD proteins (large *ε *) decrease the oxygen-tension at which FIH-release occurs (Figure 5C, left hand panel). Consequently, if *ε *is large, FIH is already released at oxygen-tensions at which PHD-activity is still only moderate, and a substantial amount of HIFα is available for CAD-hydroxylation, leading to increased peak values of CAD-hydroxylated HIFα and a sharper response (Figure 5C, right hand panel). The exact value of *ε *then determines both the degree of hypoxia at which HIFα CAD-hydroxylation can occur, and the extent to which CAD-hydroxylated HIFα can accumulate.

**Figure 6:**
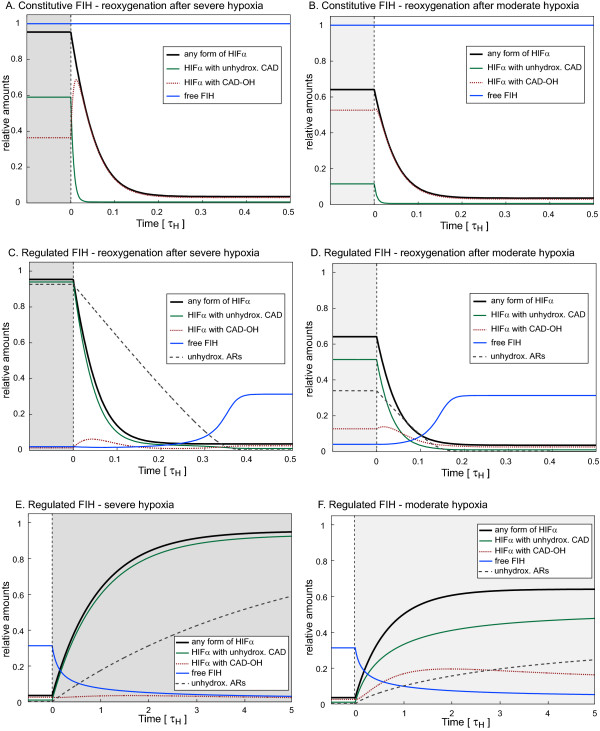
**Time course simulations in the Full Model**. A-D. Response to reoxygenation. Reoxygenation in the hypothetical case of constitutively active FIH that is not affected by competing ARs (A and B) or in the case of an FIH/AR-interaction (C and D), from either severe hypoxia (${\tilde{O}}_{2}$ = 0.001, A and C, dark grey area) or moderate hypoxia (${\tilde{O}}_{2}$ = 0.01, B and D, light grey area) to normoxic conditions (${\tilde{O}}_{2}$ = 0.5) at *t *= 0. E, F. Response to hypoxia in the case of an FIH/AR-interaction. At *t *= 0, oxygen levels were changed from normoxic (${\tilde{O}}_{2}$ = 0.5) to either severely hypoxic (${\tilde{O}}_{2}$ = 0.001, E, dark grey area) or moderately hypoxic (${\tilde{O}}_{2}$ = 0.01, F, light grey area). For completion, the behaviour in response to the same changes in oxygen levels but in the hypothetical absence of an FIH/AR-interaction is shown in Additional File 3, Figure S3. Parameters were *A_tot _*= 100, *γ *= 0.02 and *ε *= 5 for all of Figures 6 and S3. Time is given in units of the mean life time of HIFα in the absence of oxygen (*τ*_H_).

In addition to these steady state calculations, we also used the Full Model to simulate the effect of the FIH/AR-interaction on the temporal behaviour of the system in response to both hypoxia and to reoxygenation after hypoxia. We compared the hypothetical case of constitutive FIH activity (no FIH/AR-interaction) with the situation where FIH activity is regulated by ARs through sequestration and oxygen-dependent release. The simulations were performed for two different degrees of hypoxia, severe and moderate. In the case of constitutively active FIH, reoxygenation from severe hypoxia is predicted to cause a strong and transient burst in HIFα CAD-hydroxylation at early time points after reoxygenation (Figure 6A, red curve), which is attenuated if the preceding hypoxia was moderate (Figure 6B, red curve). Irrespective of the strength of the preceding hypoxic episode, in the absence of an FIH/AR-interaction nearly all HIFα is predicted to become CAD-hydroxylated prior to its oxygen-dependent degradation (Figures 6A, B). In the presence of an FIH/AR-interaction by contrast, the Full Model predicts the existence of a temporal threshold for FIH-release, which now occurs with a time delay in a switch-like fashion (Figures 6C and 6D, blue curves). These results are consistent with the predictions by SKM2 (Figure 4E, F). As a consequence of the delayed FIH-release, and in stark contrast to the case of constitutive FIH activity, the Full Model predicts that nearly all HIFα is degraded before its CAD can be hydroxylated (Figure 6C and 6D), which precludes the burst of HIFα CAD-hydroxylation upon reoxygenation seen in the case of constitutively active FIH (compare Figures 6A and 6C, red curves). The length of the delay in FIH-release depends on the hydroxylation status of the ARD protein pool. Because more severe, or longer, hypoxia leads to a higher fraction of FIH-binding ARs to be unhydroxylated, more time is required for FIH to hydroxylate these substrates and to trigger its own release (compare Figures 6C and 6D, blue curves and black dotted curves). The delay in FIH-release thus is a direct readout of the hydroxylation status of the ARD protein pool, which in turn reflects both the strength and the duration of the preceding hypoxic episode. This idea relies on the following model predictions: Firstly, the rate of accumulation of unhydroxylated ARs during hypoxia is counteracted by residual FIH activity and is thus dependent on the hypoxic oxygen tension (compare Figures 6E and 6F, slope of the dotted curves). Secondly, because Asn-hydroxylation is irreversible, the replacement of hydroxylated ARD proteins with their unhydroxylated counterparts during hypoxia is a slow process limited by *de novo *protein synthesis (compare the reoxygenation time scale in Figures 6A-D with the hypoxia timescale in Figures 6E, F). Due to the slow accumulation of unhydroxylated ARD proteins in hypoxia, which additionally depends on the residual FIH-hydroxylation activity and thus the degree of hypoxia, the hydroxylation status of the ARD protein pool can measure and integrate a wide range of hypoxic strengths and durations. At the time of reoxygenation, the hydroxylation status of the ARD protein pool will reflect these characteristics of the hypoxic episode, which then translate into a delay for FIH-release. For completion, the temporal behaviour in response to hypoxia in the absence of an FIH/AR-repeat interaction is shown in the Additional File 3, Figure S3.

In summary, steady-state simulations with the Full Model suggest that, in the absence of other variables, a low binding affinity of FIH for already hydroxylated ARs, as well as a fast basal turnover of HIFα compared to the average turnover of ARD proteins are essential requirements for efficient HIFα CAD-hydroxylation. The model predicts two functionally significant effects of oxygen-dependent FIH-release from ARD proteins, which are emergent system properties that shape the hypoxic response: The process of HIFα CAD-hydroxylation is focused into a defined range of oxygen-tensions (range-finding mechanism), and the signal/response curves are significantly sharpened (ultrasensitivity). Time course simulations further suggest that a delay in FIH-release in response to reoxygenation provides a readout of the hydroxylation status of the ankyrin-pool, which in turn encodes or "memorises" duration and strength of the preceding hypoxic episode.

## Discussion

Several kinetic models of the HIF-pathway have been reported, each focusing on different aspects of this signalling system [35-38]. Only one of these models considers FIH and HIFα CAD-hydroxylation, but this does not include ARD proteins as competing FIH substrates [35]. Predictions derived from SKM1, which ignores the FIH/AR-interaction, support previous experimental [7] and theoretical work [35]. Despite its simplicity, SKM1 successfully reproduces the findings that, first, expression of CAD-dependent genes requires more severe hypoxia when compared to NAD-dependent genes, and second, that higher FIH activity increases the magnitude of this shift towards severe hypoxia (Figure 3). These features are generic properties that are valid whether or not the FIH/AR interaction is taken into account. Our proposal that there is an additional group of target genes that relies on the hydroxylated CAD for transcriptional activation in a CBP/P300-independent manner provides a possible explanation for the unusual activation profile of HIF target genes such as *BNIP3 *[7]. The existence of such a target gene group however requires experimental verification. Because FIH needs to compete with PHD-induced degradation of HIFα, SKM1 predicts that HIFα CAD-hydroxylation can only occur to a significant extent if FIH-dependent hydroxylation is at least as effective as PHD-dependent hydroxylation (see *α *= 1 in Figure 3A for identical activities of FIH and PHDs). This is true even in the hypothetical absence of an inhibitory FIH/AR-interaction, i.e. if FIH-activity towards HIFα is constitutive and maximal. Indeed, FIH has been suggested to be a more efficient enzyme than the PHDs because of its lower K_M _for oxygen, for which there is *in vitro *evidence [30]. Our SKM1 predictions modify this notion, indicating that efficient HIFα CAD-hydroxylation does not specifically depend on the K_M _of FIH for oxygen, but more generally on intracellular FIH activity. Expression levels of FIH relative to PHDs thus are predicted to play a major role *in vivo *in determining the extent of HIFα CAD-hydroxylation at a given oxygen tension, and accurate measurements of relative expression levels of FIH and PHDs are desirable.

The focus of our work was on the regulation of FIH activity towards HIFα through competitive inhibition by ARD proteins. The large number of ARD proteins, a substantial fraction of which we predict to be FIH targets, and the fact that at least some isolated ankyrin-domains bind more tightly to FIH than to HIFα [13,39] make it seem possible that ARs will out-compete HIFα and prevent significant HIFα CAD-hydroxylation irrespective of the oxygen concentration. However, HIFα clearly is CAD-hydroxylated by FIH *in vivo*, and a conclusive theory needs to consolidate this fact with the presence and the action of FIH-binding ARD proteins. Several factors can potentially contribute to weakening the competition by ARD proteins for FIH. First, not all ARD proteins are expected to be expressed in a particular cell type. Second, the ARD is a protein-protein interaction motif, and only a fraction of a given ARD protein is expected to be accessible to FIH *in vivo*. Third, affinity constants measured *in vitro *with isolated protein domains might be misleading. These considerations make it difficult to give an estimate of a realistic concentration of FIH binding target repeats, and FIH sequestration could be much less pronounced than expected from idealised theoretical considerations. Irrespective of its actual extent however, FIH sequestration by ARD proteins must be oxygen-regulated in order to tune HIFα CAD-hydroxylation, other than just repressing it at all oxygen tensions, and experimental evidence points to hydroxylation-dependent release of FIH from ARD-proteins as the responsible mechanism. SKM2 predicts that such FIH-release only occurs if hydroxylation of FIH-accessible ARs approaches completion. Importantly, this prediction is not in contrast with experimental findings indicating that the hydroxylation of an individual AR is often far from complete, even under conditions of high oxygen [11,13,15]. Because the ARD is a protein-protein interaction domain, the access of FIH to the ARDs will be restricted by the presence of ARD-interactors other than FIH, which will partly protect ankyrin repeats from FIH-dependent hydroxylation. Thus, it is possible that near-complete hydroxylation of a specific AR may not be observed experimentally, but the hydroxylation of its FIH-accessible fraction might still approach completion.

We identify several potential functional effects of the FIH/AR-interaction on HIFα CAD-hydroxylation. The Full Model predicts that the FIH/AR-interaction introduces an oxygen-threshold, below which HIFα CAD-hydroxylation is marginal and CAD-dependent genes are fully active. The exact threshold concentration of oxygen, above which HIFα CAD-hydroxylation can occur and CAD-dependent genes are turned off, is predicted to be determined by the total amount of FIH-accessible, hydroxylatable ARs, their binding affinity for FIH, as well as by the relative turnover rates of HIFα and ARD proteins and should thus be tunable by modulating these system features. A range finding property is essential for the HIF hydroxylases to act as oxygen sensors in environments with different physiologically relevant ranges of oxygen concentrations [1]. Although it is unlikely that the FIH/AR-interaction is the only range-finding mechanism in the HIF system, our results demonstrate that it is one possible such mechanism. The change in CAD-hydroxylation at the oxygen threshold is significantly sharpened compared to the absence of an FIH/AR-interaction. Such ultrasensitivity, as characterised by a sigmoid signal/response curve, is an important feature of many signalling pathways and cellular decision making processes. Mechanisms giving rise to ultrasensitive behaviour include cooperativity, multi-site modification, zero-order ultrasensitivity, and positive feedback. In our case, the mechanism causing ultrasensitivity is similar to a proposed "ultrasensitivity by substrate competition", where stoichiometric inhibition of an enzyme by a competing substrate can make enzyme activity for other substrates nonlinearly dependent on the enzyme level [40]. Finally, time course simulations using SKM2 (Figure 4) and the Full Model (Figure 6) predict that FIH-release can occur in a switch-like fashion with a time delay after reoxygenation of hypoxic cells. The length of this delay depends on the intensity and the duration of the preceding hypoxic period. Functionally, this time delay in FIH-release can prevent a premature FIH-release during a brief and perhaps only transient reoxygenation event, and only sustained reoxygenation will trigger bulk FIH-release.

## Conclusions

Overall, the combined modelling results reveal that the interaction of multiple ARD proteins with FIH has the potential to significantly input on the dynamics of the hypoxic response in human cells. The FIH/AR-interaction can provide a mechanism by which the oxygen-threshold for the hypoxic response can be varied (range finding mechanism), it can confer substantial sharpening of the signal/response curves (ultrasensitivity), and it can create a time-delay for CAD-hydroxylation after reoxygenation, the length of which can encode the strength and duration of the preceding hypoxic episode (a memory effect). The modulation of HIF hydroxylase activity is of considerable interest with respect to therapeutic intervention. Inhibition of the HIF hydroxylases may be useful for treatment of anaemia and ischemic disease, via the upregulation of erythropoiesis and angiogenesis, respectively. Presently, it is unclear whether HIF hydroxylase inhibitors should target individual HIF hydroxylases or combinations of enzymes. Our results reveal that the FIH/AR-interactions should be taken into account in analyses of the cellular and physiological effects of HIF hydroxylase inhibitors, whether or not these inhibitors are selective for PHDs and/or FIH. Interfering with the binding of FIH to ARD-proteins is predicted to increase HIFα CAD-hydroxylation, whereas interfering with AR-hydroxylation by FIH is predicted to decrease HIFα CAD-hydroxylation. Thus, the FIH/AR-interaction itself is a promising potential target for pharmacological modulation of the HIF pathway. Finally, we note that if the concept of multiple proteins regulating a signalling sensor occurs in the oxygen sensing HIF-system, it is likely to occur in other signalling pathways.

## List of Abbreviations Used

AR: ankyrin repeat; ARD: ankyrin repeat domain; CAD: *C*-terminal transactivation domain; CADOH: Asn-hydroxylated C-terminal transactivation domain; DMOG: dimethyloxalylglycine; EGLN: egl nine homolog; FIH: factor inhibiting HIF; HIF: hypoxia inducible factor; HRE: hypoxia response element; NAD: *N*-terminal transactivation domain; ODD: oxygen-dependent degradation domain; PHD: prolyl hydroxylase domain.

## Authors' contributions

BS carried out the bioinformatic analysis, devised and analysed the kinetic models and prepared the figures. BN participated in the design of the modelling and CJS initiated the study. All authors contributed to writing the manuscript and approved its final version.

## Supplementary Material

Additional file 1 **A database of human ankyrin repeats**. Click here for file

Additional file 2 **Supplementary Methods**. Description of the Full Model kinetics (Section 1) and detailed derivation of the hydroxylation rate functions *ν_P_*, *ν_FH _*and *ν_FA _*(Sections 2-4), non-dimensionalisation of the Full Model (section 5) and of Skeleton Model 2 (Section 6), as well as references used in this file. Click here for file

Additional file 3 **Supplementary Table and Figures**. Table S1, Table S2 and Supplementary Figures S1, S2, S3, S4. Click here for file

Additional file 4 **The XPP-AUT model file**. Running this code in XPP-AUT produces Figure 5B, left and right hand panels for a specific parameter set. Click here for file
