# Supplementary material for: Hypoxia-dependent sequestration of an oxygen sensor by a widespread structural motif can shape the hypoxic response - a predictive kinetic model
Source: BMC Syst Biol. 2010 Oct 18;4:139. doi: 10.1186/1752-0509-4-139 (PMC2984394; doi:10.1186/1752-0509-4-139)
Supplement: Additional file 2 — Supplementary Methods. Description of the Full Model kinetics (Section 1) and detailed derivation of the hydroxylation rate functions νP, νFH and νFA (Sections 2-4), non-dimensionalisation of the Full Model (section 5) and of Skeleton Model 2 (Section 6), as well as references used in this file. [file 1752-0509-4-139-S2.PDF]

ADDITIONAL FILE 2. SUPPLEMENTARY METHODS.

1. The hydroxylation rate functions.

Michaelis-Menten kinetics are only strictly valid if the substrate is in excess over the enzyme, and if formation of enzyme-substrate complexes does not significantly decrease the concentration of free substrate [1]. In protein-protein interaction networks, this is not necessarily true. In our case, the concentration of the substrate HIFα can become very low, and cannot be assumed to be in excess over the hydroxylases at all times. Thus, we model the HIFα hydroxylation reactions by taking into account that free HIFα is decreased by complex formation with the hydroxylases. Figure S4 compares the results obtained by this method with a Michaelis-Menten approximation. Section 2 derives the expressions for PHD-dependent ODD-hydroxylation in detail. HIFα CAD-hydroxylation by FIH in the presence of competing ARD proteins (Section 3) as well as AR-hydroxylation (Section 4) follow the same scheme. All expressions obtained reduce to simple Michaelis-Menten-type kinetics if substrate concentrations are large compared to enzyme concentrations.

2. Derivation of a rate function for HIFα ODD-hydroxylation.

There is strong evidence that oxygen and HIFα can bind to hydroxylases independently of each other [2], which gives rise to the following reaction scheme. H_P indicates all forms of HIFα that can bind to PHD (i.e. are not already bound to PHD). P_0 is PHD not bound by either HIFα or oxygen, and P_1, P_2, P_{12} indicate complexes of PHD bound to oxygen, HIFα, or both, respectively.

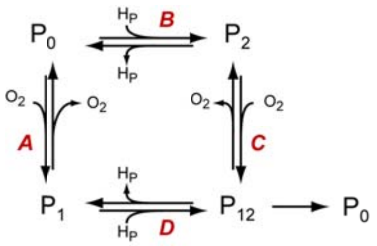

A. $P_0 + O_2 \leftrightarrows P_1$

B. $P_0 + H_P \leftrightarrows P_2$

C. $P_2 + O_2 \leftrightarrows P_{12}$

D. $P_1 + H_P \leftrightarrows P_{12}$

Catalysis: $P_{12} \rightarrow P_0 + H_{OH}^P \rightarrow P_0$

ODD-hydroxylated HIF is very unstable and, as a first approximation, degraded instantaneously [3]. The hydroxylation rate can thus be viewed as a degradation rate and is equal to the catalytic turnover of the productive complex, P_{12}, for which we need to derive an expression. The change of P_{12} with time is given by the differential equation (2.1), where k_{on} and k_{off} are the on- and off-rate constants for oxygen binding, and k'_{on} and k'_{off} for HIFα binding, respectively.

$$\frac{dP_{12}}{dt} = k_{on}P_2O_2 + k'_{on}P_1H_p - P_{12}(k_{off} + k'_{off} + k^P_{cat}) \qquad 2.1$$

Binding of HIFα to PHD has been suggested to be fast compared to the enzyme's reaction with oxygen [4], and we treat binding reactions B and D as at steady state. In this case,

$$k'_{on}P_1H_p = k'_{off}P_{12} \qquad 2.2$$

and (2.1) simplifies to (2.3), where K_M^P is the Michaelis constant of PHD for oxygen. From this equilibrium assumption, we also obtain (2.4) and (2.5), where K_D^P indicates the dissociation constant of the PHD/ HIFα complex.

$$\frac{dP_{12}}{dt} = k_{on}(P_2O_2 - K_M^P P_{12}) \qquad 2.3$$

$$P_2 = P_0\frac{H_P}{K_D^P} \qquad 2.4$$

$$P_{12} = P_1\frac{H_P}{K_D^P} \qquad 2.5$$

The total amount of PHD, P_{tot}, is conserved, and with (2.4) and (2.5) given as

$$P_{tot} = P_0 + P_1 + P_2 + P_{12} = (P_0 + P_1)\frac{K_D^P + H_P}{K_D^P} \qquad 2.6$$

If the productive complex P_{12} is at steady state, (2.3) equals zero, and with (2.3) and (2.4) we obtain

$$P_{12} = P_0\frac{O_2}{K_M^P}\frac{H_P}{K_D^P} \qquad 2.7 \qquad\qquad P_1 = P_0\frac{O_2}{K_M^P} \qquad 2.8$$

Combining (2.7), with (2.5) yields (2.8), which we substitute into (2.6) to obtain (2.9), which, with (2.7) yields the expression for the productive complex (2.10) and thus the hydroxylation (=degradation) rate (2.11).

$$P_0 = P_{tot}\left(\frac{K_D^P}{K_D^P + H_P}\right)\left(\frac{K_M^P}{K_M^P + O_2}\right) \qquad 2.9$$

$$P_{12} = P_{tot}\left(\frac{H_P}{K_D^P + H_P}\right)\left(\frac{O_2}{K_M^P + O_2}\right) \qquad 2.10$$

$$-\frac{dH_{tot}}{dt} = k_{cat}^P\, P_{tot}\left(\frac{H_P}{K_D^P + H_P}\right)\left(\frac{O_2}{K_M^P + O_2}\right) \qquad 2.11$$

In the classical Michaelis-Menten approximation, the amount of substrate bound to enzyme is considered negligible and H_P is replaced by H_{tot}, the total amount of HIFα present in the system. Using (2.6), we obtain H_P as an explicit function of H_{tot} from mass conservation:

$$H_{tot} = H_P + P_2 + P_{12} = H_P + P_{tot}\frac{H_P}{K_D^P + H_P} \qquad 2.12$$

$$H_P^2 + H_P\,(K_D^P + P_{tot} - H_{tot}) - K_D^P H_{tot} = 0 \qquad 2.13$$

$$H_P = \frac{1}{2}\left(H_{tot} - P_{tot} - K_D^P + \sqrt{(K_D^P + P_{tot} - H_{tot})^2 + 4K_D^P H_{tot}}\right) \qquad 2.14$$

The given solution is the biologically relevant of the two roots of the quadratic equation. Finally, we rewrite (2.12) to obtain (2.15), which we combine with (2.11) to obtain our final expression for the hydroxylation and thus degradation

rate (2.16). Division by H_{tot} gives the rate function v_P for HIFα hydroxylation by PHD (2.17), which we will use in the system of ODEs.

$$H_P = H_{tot} \frac{K_D^P + H_P}{K_D^P + H_P + P_{tot}} \qquad 2.15$$

$$-\frac{dH_{tot}}{dt} = k_{cat}^P P_{12} = k_{cat}^P\, P_{tot} \left(\frac{H_{tot}}{K_D^P + H_P + P_{tot}}\right)\left(\frac{O_2}{K_M^P + O_2}\right) \qquad 2.16$$

$$v_P = k_{cat}^P\, P_{tot} \left(\frac{1}{K_D^P + H_P + P_{tot}}\right)\left(\frac{O_2}{K_M^P + O_2}\right) \qquad 2.17$$

It is immediately clear from (2.15) that, for small enzyme concentrations, $H_P \cong H_{tot}$, in this case the rate equation (2.16) becomes a classical Michaelis-Menten-type function (2.18). Expressions of this form are used in Skeleton Models 1 and 2.

$$-\frac{dH_{tot}}{dt} = k_{cat}^P P_{12} = k_{cat}^P\, P_{tot} \left(\frac{H_{tot}}{K_D^P + H_{tot}}\right)\left(\frac{O_2}{K_M^P + O_2}\right) \qquad 2.18$$

3. Derivation of a rate function for HIFα CAD-hydroxylation in the presence of ARD proteins.

The binding and hydroxylation reactions for FIH are:

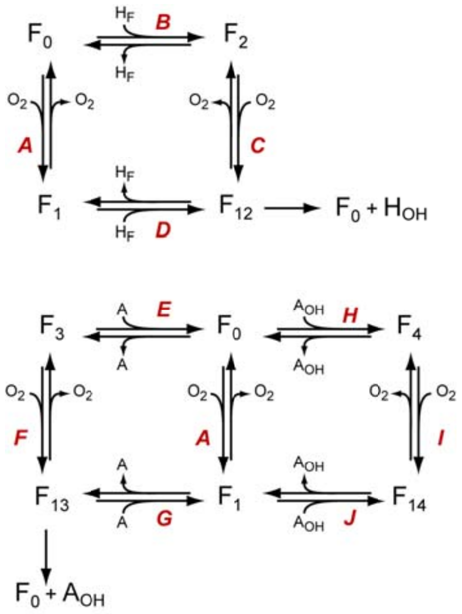

A.	$F_0 + O_2 \leftrightarrows F_1$		F.	$F_3 + O_2 \leftrightarrows F_{13}$
B.	$F_0 + H_F \leftrightarrows F_2$		G.	$F_1 + A \leftrightarrows F_{13}$
C.	$F_2 + O_2 \leftrightarrows F_{12}$		H.	$F_0 + A_{OH} \leftrightarrows F_4$
D.	$F_1 + H_F \leftrightarrows F_{12}$		I.	$F_4 + O_2 \leftrightarrows F_{14}$
E.	$F_0 + A \leftrightarrows F_3$		J.	$F_1 + A_{OH} \leftrightarrows F_{14}$

Catalysis
$F_{12} \rightarrow F_0 + H_{OH}$
$F_{13} \rightarrow F_0 + A_{OH}$

Here, H_F is all HIF that is not CAD-hydroxylated and not bound to FIH, and the H_{OH} is the reaction product, CAD-hydroxylated HIFα. Free FIH (F_0) can bind its substrates oxygen and HIFα in arbitrary order to form the productive complex F_{12}, the same is true for oxygen and unhydroxylated ARs (A) to form the productive complex F_{13}. FIH can also bind weakly to hydroxylated ARs (A_{OH}). Such binding causes sequestration of FIH in the complexes F_4 and F_{14}, but does not yield a productive complex. Note that binding of one protein substrate to FIH precludes binding of any other protein substrate to the enzyme. Because ARs are in excess over FIH, we neglect substrate depletion by binding to FIH for ARs and use the classical Michaelis-Menten approximation, i.e. we replace the free forms of unhydroxylated and hydroxylated ARs by the total amounts.

As in the previous derivation of HIFα ODD-hydroxylation, we assume that enzyme binding to the protein substrates is fast, and that the corresponding reactions (B, D, E, G, H, J) are at steady state. We obtain in analogy to (2.6)

$$F_{tot} = F_0 + F_1 + F_2 + F_{12} + F_3 + F_{13} + F_4 + F_{14} =$$
$$= (F_0 + F_1)\frac{K_D^{FH}\left(1 + \frac{A}{K_D^{FA}} + \frac{\gamma A_{OH}}{K_D^{FA}}\right) + H_F}{K_D^{FH}} \qquad 3.1$$

K_D^{FH} and K_D^{FA} are the dissociation constants of the FIH/HIFα- and FIH/AR-interaction, respectively. $\gamma < 1$ is the factor by which affinity of ankyrin repeats for FIH decreases by hydroxylation. By defining K_i^{FH} (3.2) and by using an analogous derivation to (2.1 – 2.8), we obtain (3.3), which is of the same form as (2.9).

$$K_D^{FH}\left(1 + \frac{A}{K_D^{FA}} + \frac{\gamma A_{OH}}{K_D^{FA}}\right) \stackrel{\text{def}}{=} K_i^{FH} \qquad 3.2$$

$$F_0 = F_{tot}\left(\frac{K_D^{FH}}{K_i^{FH} + H_F}\right)\left(\frac{K_M^F}{K_M^F + O_2}\right) \qquad 3.3$$

From a derivation analogous to (2.10) – (2.17), we obtain our final expressions for the HIFα CAD-hydroxylation rate (3.4) and the corresponding rate function v_{FH} (3.5) in the presence of competing ankyrin repeats:

$$\frac{dH_{OH}}{dt} = k_{cat}^{FH} F_{12} = k_{cat}^{FH}\, F_{tot}\left(\frac{H}{K_i^{FH} + H_F + F_{tot}}\right)\left(\frac{O_2}{K_M^F + O_2}\right) \qquad 3.4$$

$$v_{FH} = k_{cat}^{FH}\, F_{tot}\left(\frac{1}{K_i^{FH} + H_F + F_{tot}}\right)\left(\frac{O_2}{K_M^F + O_2}\right) \qquad 3.5$$

$$H_F = \frac{1}{2}\left(H - F_{tot} - K_i^{FH} + \sqrt{(K_i^{FH} + F_{tot} - H)^2 + 4K_i^{FH}H}\right) \qquad 3.6$$

H indicates the total amount of HIFα that is not CAD-hydroxylated. In the absence of competitive inhibition by ARD proteins, HIFα CAD-hydroxylation is given by expressions of identical forms to (3.4 – 3.5), but with K_i^{FH} replaced by K_D^{FH}. By substituting for K_i^{FH} from (3.2) in the case of $FIH >> H_F$ so that $H_F \cong H$, we see that the HIF-term in (3.4) reduces to the classical form of competitive inhibition:

$$\frac{H}{H + K_D^{FH} + \frac{K_D^{FH}}{K_D^{FA}}(A + \gamma A_{OH})} = \frac{[S]}{[S] + K_M + \frac{K_M}{K_I}[I]} \qquad 3.7$$

4. The rate function for Asn-hydroxylation of ankyrin repeats.

Equivalently to (3.3) and using the definition (4.1), F_0 can also be expressed as (4.2), and we obtain the ankyrin hydroxylation rate (4.3) and the corresponding rate function v_{FA} (4.4) in the presence of competing HIFα:

$$K_D^{FA}\left(1 + \frac{H_F}{K_D^{FH}}\right) \stackrel{\text{def}}{=} K_i^{FA} \qquad 4.1$$

$$F_0 = F_{tot}\left(\frac{K_D^{FA}}{K_i^{FA} + A + \gamma A_{OH}}\right)\left(\frac{K_M^F}{K_M^F + O_2}\right) \quad 4.2$$

$$\frac{dA_{OH}}{dt} = k_{cat}^{FA}F_{13} = k_{cat}^{FA}\,F_{tot}\left(\frac{A}{K_i^{FA} + A + \gamma A_{OH}}\right)\left(\frac{O_2}{K_M^F + O_2}\right) \quad 4.3$$

$$\nu_{FA} = k_{cat}^{FA}\,F_{tot}\left(\frac{1}{K_i^{FA} + A + \gamma A_{OH}}\right)\left(\frac{O_2}{K_M^F + O_2}\right) \quad 4.4$$

As for the HIF term in (3.4) where ankyrin repeats were the inhibitors, the ankyrin term in (4.3) reduces to classical competitive inhibition if $F_{tot} >> H_F$, but now HIFα is the competitive inhibitor. Finally, to obtain an explicit expression for FIH not bound to ARD proteins, F_{free}, we use (4.6), which with (3.1), gives the amount of free FIH (4.7).

$$F_{free} = F_0 + F_1 + F_2 + F_{12} = (F_0 + F_1)\,\frac{K_D^{FH} + H_F}{K_D^{FH}} \quad 4.6$$

$$F_{free} = F_{tot}\,\frac{K_D^{FH} + H_F}{K_i^{FH} + H_F} \quad 4.7$$

Figure S4 compares a simulation using the full model with a simulation using Michaelis-Menten kinetics. Because the concentration of the PHDs is assumed low compared to HIFα, there is not much difference in the levels of total HIFα (black curves). The excess of FIH compared to HIFα however cause the results to differ more substantially, with the full model giving lower levels of CAD-hydroxylated HIFα. Moreover, the peak is reached at a higher oxygen concentration (compare red curves). While the differences do not affect any of the conclusions in the present work, the approach we have introduced here will be important for future, more quantitative models of HIFα hydroxylation.

5. The Full Model and its non-dimensionalisation.

The full model is given by three differential equations, one each for total HIFα (H_{tot}), HIFα that is not CAD-hydroxylated (H), and one for unhydroxylated AR (A). The concentrations of CAD-hydroxylated HIFα (H_{OH}) and hydroxylated AR (A_{OH}) are given by mass conservation of the total amounts, H_{tot} and A_{tot}.

$$\frac{dH_{tot}}{dt} = k_s^H - H_{tot}\,(k_d^H + \nu_P) \quad 5.1$$

$$\frac{dH}{dt} = k_s^H - H\,(k_d^H + \nu_P + \nu_{FH}) \quad 5.2$$

$$\frac{dA}{dt} = k_s^A - A\,(k_d^A + \nu_{FA}) \quad 5.3$$

$$H_{OH} = H_{tot} - H \quad 5.4$$

$$A_{OH} = A_{tot} - A \quad 5.5$$

k_s and k_d are the basal protein synthesis and degradation rates, respectively, for the species indicated by superscript. We non-dimensionalise the system of ODEs by normalising to the maximally possible amount of HIFα, and by scaling time with the basal degradation rate constant of HIFα. Thus, with

$$H_{tot}^{max} = \frac{k_s^H}{k_d^H} \qquad d\tau = k_d^H dt \qquad \varepsilon = \frac{k_d^H}{k_d^A} = \frac{\tau_A}{\tau_H}$$

we obtain Eq. 1 – 3 given in the main text. where "hat" (^) indicates non-dimensional quantities expressed relative to H_{tot}^{max}, and "prime" (') indicates non-dimensional quantities expressed relative to k_d^H. The parameter ε is the half life ratio of ARD proteins and HIFα under basal turnover conditions, i.e. in the absence of oxygen. We introduce

$$\tilde{O}_2 = \frac{O_2}{K_M^P} \qquad \alpha = \frac{K_M^F}{K_M^P}$$

and express the hydroxylation rate functions $\acute{\nu}_P$, $\acute{\nu}_{FH}$ and $\acute{\nu}_{FA}$ as functions of the new non-dimensional variables to obtain the expressions given in the main text (Eq. 4, 6 and 9).

6. Skeleton Model 2 and its non-dimensionalisation.

If we assume, as an approximation to experimental data [5], that FIH does only bind to unhydroxylated but not hydroxylated AR ($\gamma = 0$) and we ignore the presence of HIFα, we can describe AR-hydroxylation by the differential equation (6.1), which is a simplified version of (5.3). FIH not bound to AR is given by (6.2), which is obtained by employing these assumptions to (4.7).

$$\frac{dA}{dt} = k_s^A - k_d^A A - k_{cat}^{FA}\,F_{tot}\,\frac{A}{K_D^{FA} + A}\,\frac{O_2}{K_M^F + O_2} \quad 6.1$$

$$F_{free} = F_{tot}\,\frac{K_D^{FA}}{K_D^{FA} + A} \quad 6.2$$

With the definitions

$$d\sigma = k_d^A dt \qquad \hat{A} = \frac{A\,k_d^A}{k_s^A} \qquad \beta = \frac{k_{cat}^{FA}F_{tot}}{k_d^A A_{tot}}$$

$$\bar{O}_2 = \frac{O_2}{K_M^F} \qquad \hat{F}_{free} = \frac{F_{free}\,k_d^A}{k_s^A} = \frac{F_{free}}{A_{tot}} \qquad \kappa = \frac{k_s^A}{K_D^{FA}\,k_d^A} = \frac{A_{tot}}{K_D^{FA}}$$

(6.6) and (6.7) can be written in the non-dimensional form given in the main text.

7. References for Additional File 2, Supplementary Methods.

1. Borghans JA, de Boer RJ, Segel LA: **Extending the quasi-steady state approximation by changing variables.** *Bull Math Biol* 1996, **58:**43-63.

2. Hausinger RP: **FeII/alpha-ketoglutarate-dependent hydroxylases and related enzymes.** *Crit Rev Biochem Mol Biol* 2004, **39:**21-68.

3. Kaelin WG, Jr., Ratcliffe PJ: **Oxygen sensing by metazoans: the central role of the HIF hydroxylase pathway.** *Mol Cell* 2008, **30:**393-402.

4. Flashman E, Davies SL, Yeoh KK, Schofield CJ: **Investigating the dependence of the hypoxia-inducible factor hydroxylases (factor Inhibiting HIF and prolyl hydroxylase domain 2) on ascorbate and other reducing agents.** *Biochem J* 2010.

5. Coleman ML, McDonough MA, Hewitson KS, Coles C, Mecinovic J, Edelmann M, Cook KM, Cockman ME, Lancaster DE, Kessler BM, et al: **Asparaginyl hydroxylation of the Notch ankyrin repeat domain by factor inhibiting hypoxia-inducible factor.** *J Biol Chem* 2007, **282:**24027-24038.
