# Supplementary material for: Hypoxia-dependent sequestration of an oxygen sensor by a widespread structural motif can shape the hypoxic response - a predictive kinetic model
Source: BMC Syst Biol. 2010 Oct 18;4:139. doi: 10.1186/1752-0509-4-139 (PMC2984394; doi:10.1186/1752-0509-4-139)
Supplement: Additional file 3 — Supplementary Table and Figures. Table S1, Table S2 and Supplementary Figures S1, S2, S3, S4. [file 1752-0509-4-139-S3.PDF]

ADDITIONAL FILE 3. Supplementary Table and Figures.

Table S1. Verified FIH targets.

Name	Full Protein Name	Uniprot Accession	Sequence motif	Position of Asn-OH	Experimental Conditions	References
**MYPT1**	Protein phosphatase 1 regulatory subunit 12A	O14974	**L**LHRGADI**N**YANV	67	In vivo, endogenous proteins	[1]
			**L**IQAGYDV**N**IKDY	226		
			**L**VENGANI**N**QPDN	100		
NOTC1	NOTCH-1	P46531	**L**LEASADA**N**IQDN	1955		[2]
IKBA	NF-kappa-B inhibitor alpha	P25963	**L**LKCGADV**N**RVTY	244		[3]
			**L**VSLGADV**N**AQEP	210	In vivo, substrate overexpressed	
NFKB1	Nuclear factor NF-kappa-B p105 subunit	P19838	**L**VAAGADV**N**AQEQ	678		
RN5A	2-5A-dependent ribonuclease	Q05823	**L**DEMGADV**N**ACDN	196		[4]
TNKS2	Tankyrase-2	Q9H2K2	**L**LQHGADV**N**AQDK	706		
			**L**VKHGAVV**N**VADL	586		
			**L**IKYNACV**N**ATDK	739		
			VVKHEAKV**N**ALDN	427		
ANFY1	Ankyrin repeat and FYVE domain-containing protein 1	Q9P2R3	**L**LEFGANV**N**AQDA	797		
			**L**IKNGAFV**N**AATL	316	In vivo, Substrate and FIH overexpressed	
			**L**ATNGAHV**N**HRNK	485		
			**L**IRSGCDV**N**SPRQ	752		
ASB4	Ankyrin repeat and SOCS box protein 4	Q9Y574	**L**LDYKAEV**N**ARDD	246		[5]
ANK1	Ankyrin-1	P16157	**L**VNYGANV**N**AQSQ	105	In vitro, peptide only	M Yang and CJ Schofield, submitted
			**L**LENGANQ**N**VATE	138		
			**L**LNRGASV**N**FTPQ	233		
			**L**LQRGASP**N**VSNV	431		
			**L**LQNKAKV**N**AKAK	464		
			**L**LQYGGSA**N**AESV	629		
			**L**LSKQANG**N**LGNK	662		
			**L**LQHQADV**N**AKTK	728		
			**L**LKNGASP**N**EVSS	761		
ANK2	Ankyrin-2	Q01484	**L**LNYGAET**N**IVTK	656		
CDN2D	Cyclin-dependent kinase 4 inhibitor D	P55273	**L**VEHGADV**N**VPDG	101		[3]
ANR49	Ankyrin repeat domain-containing protein 49	Q8WVL7	**L**LQHDADI**N**AQTK	168		
FEM1B	Protein fem-1 homolog B	Q9UK73	**L**LDCGAEV**N**AVDN	526		
GABP1	GA-binding protein subunit beta-1	Q06547	**L**LKHGADV**N**AKDM	98		
ILK	Integrin-linked protein kinase	Q13418	**L**LQYKADI**N**AVNE	94		
MTPN	Myotrophin	P58546	**L**LLKGADI**N**APDK	62		
NOTC1	NOTCH-1	P46531	**L**INSHADV**N**AVDD	2022		[2]
TNKS1	Tankyrase-1	O95271	**L**LEHGADV**N**AQDK	864		[3]
PSD10	26S proteasome non-ATPase regulatory subunit 10	O75832	**L**LGKGAQV**N**AVNQ	100		
	Ankyrin Consensus	n/a	**L**LEHGADV**N**ARDK	n/a	n/a	[6]
HIFA	Hypoxia-inducible factor 1-alpha (HIF-1α)	Q16665	**L**TSYDCEV**N**APIQ	803	Endogenous	
EPAS2	Endothelial PAS domain-containing protein 1 (HIF-2α)	Q99814	**L**TRYDCEV**N**VPVL	847		

Table S1. Ankyrin repeats that are hydroxylated by FIH either *in vivo* or *in vitro*. The ankyrin consensus sequence as well as the sequence of HIFα are given for comparison. The hydroxylated asparagine and the conserved leucine residue in position -8 relative to the asparagine are indicated in bold.

Table S2. ARD proteins that interact with FIH in a dimethyloxalylglycine- (DMOG-) inducible manner.

Name	Full Protein name	Uniprot Accession	References
UACA	Uveal autoantigen with coiled-coil domains and ankyrin repeats	Q9BZF9	[3]
NOTC2	NOTCH-2	Q04721	[2]
NOTC3	NOTCH-3	Q9UM47	
ANKH1	Ankyrin repeat and KH domain-containing protein 1	Q8IWZ3	[4]
ANR27	Ankyrin repeat domain-containing protein 27	Q96NW4	
ANR35	Ankyrin repeat domain-containing protein 35	Q8N283	
ANR52	Serine/threonine-protein phosphatase 6 regulatory ankyrin repeat subunit C	Q8NB46	
ANR60	Ankyrin repeat domain-containing protein 60	Q9BZ19	
ANS1A	Ankyrin repeat and SAM domain-containing protein 1A	Q92625	
IKBE	NF-kappa-B inhibitor epsilon	O00221	
RIPK4	Receptor-interacting serine/threonine-protein kinase 4	P57078	

Table S2. The 2-oxoglutarate analogue DMOG blocks the catalytic activity of FIH and of other 2-oxoglutarate-dependent dioxygenases. The shown ARD proteins have been found to interact with FIH in a DMOG-inducible fashion. Whether these proteins are hydroxylated by FIH is currently unclear.

References for Tables S1 and S2.

1. Webb JD, Muranyi A, Pugh CW, Ratcliffe PJ, Coleman ML: **MYPT1, the targeting subunit of smooth-muscle myosin phosphatase, is a substrate for the asparaginyl hydroxylase factor inhibiting hypoxia-inducible factor (FIH).** *Biochem J* 2009, **420:**327-333.
2. Coleman ML, McDonough MA, Hewitson KS, Coles C, Mecinovic J, Edelmann M, Cook KM, Cockman ME, Lancaster DE, Kessler BM, et al: **Asparaginyl hydroxylation of the Notch ankyrin repeat domain by factor inhibiting hypoxia-inducible factor.** *J Biol Chem* 2007, **282:**24027-24038.
3. Cockman ME, Lancaster DE, Stolze IP, Hewitson KS, McDonough MA, Coleman ML, Coles CH, Yu X, Hay RT, Ley SC, et al: **Posttranslational hydroxylation of ankyrin repeats in IkappaB proteins by the hypoxia-inducible factor (HIF) asparaginyl hydroxylase, factor inhibiting HIF (FIH).** *Proc Natl Acad Sci U S A* 2006, **103:**14767-14772.
4. Cockman ME, Webb JD, Kramer HB, Kessler BM, Ratcliffe PJ: **Proteomics-based identification of novel factor inhibiting hypoxia-inducible factor (FIH) substrates indicates widespread asparaginyl hydroxylation of ankyrin repeat domain-containing proteins.** *Mol Cell Proteomics* 2009, **8:**535-546.
5. Ferguson JE, 3rd, Wu Y, Smith K, Charles P, Powers K, Wang H, Patterson C: **ASB4 is a hydroxylation substrate of FIH and promotes vascular differentiation via an oxygen-dependent mechanism.** *Mol Cell Biol* 2007, **27:**6407-6419.
6. Mosavi LK, Minor DL, Jr., Peng ZY: **Consensus-derived structural determinants of the ankyrin repeat motif.** *Proc Natl Acad Sci U S A* 2002, **99:**16029-16034.

Figure S1

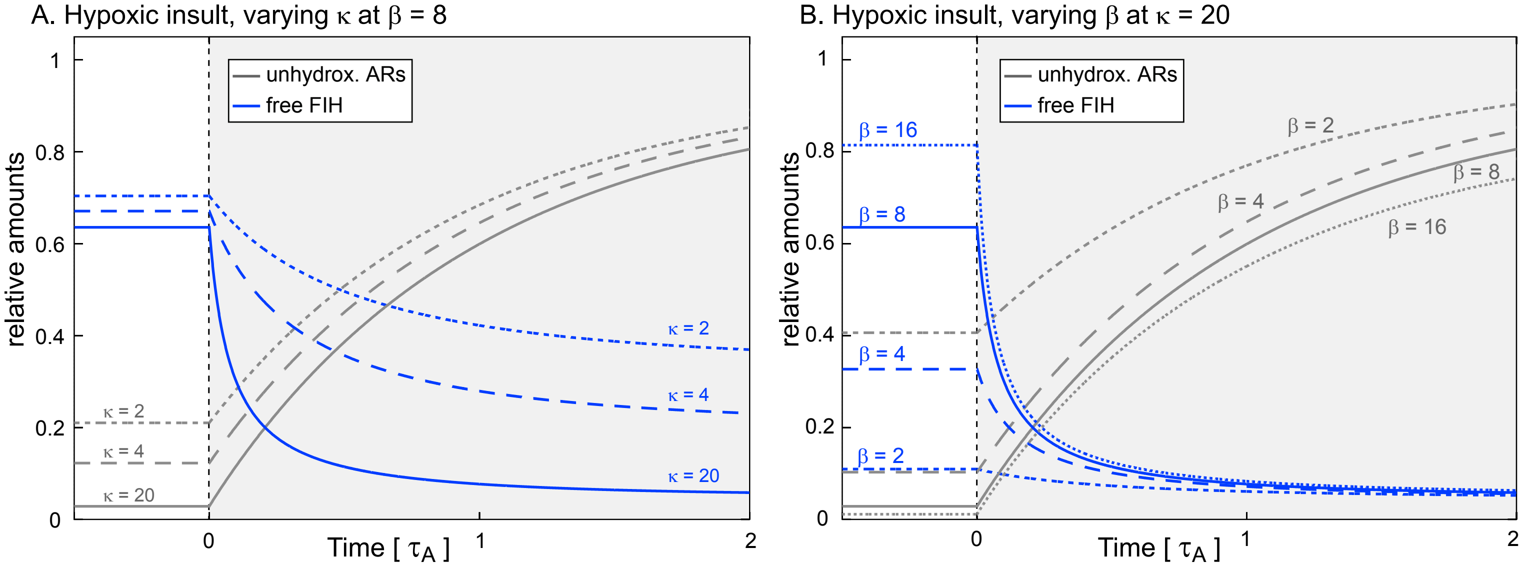

Figure S1. Skeleton Model 2. Time-resolved response to a sharp drop in oxygen. The system was equilibrated at $\bar{O}_2 = 0.5$ (white area). At $t = 0$, oxygen was decreased to $\bar{O}_2 = 0.01$ (grey area). The time-resolved response to a decrease of oxygen is rather insensitive to variation of κ and β, with the family of curves showing a hyperbolic decrease in free FIH with time, either reaching distinct steady state values at low oxygen when κ is varied (A), or starting out from distinct steady state values at high oxygen if β is varied (B). Time is given in units of the half life of an average ARD protein.

Figure S2

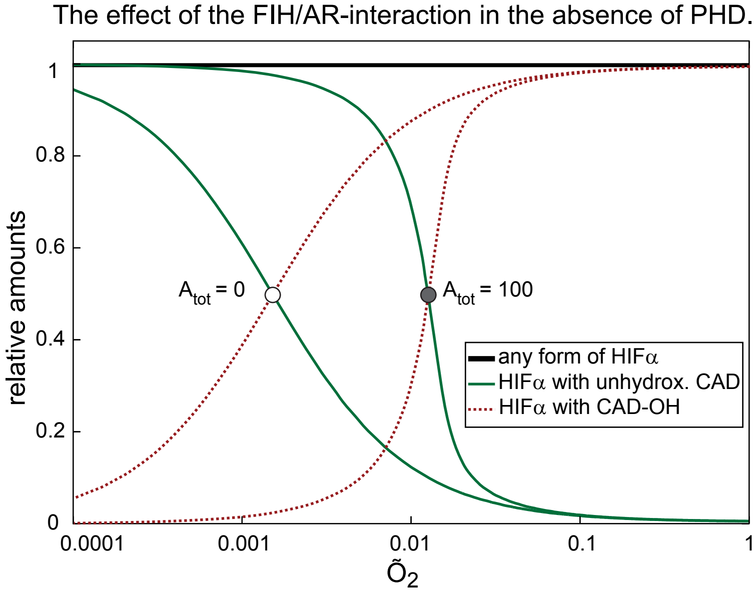

Figure S2. As Figure 5A, but in case of no PHD activity. The ultrasensitivity resulting from the FIH/AR-interaction is more obvious if there is no oxygen-dependent degradation of HIFα, in which case total HIFα levels are maximal and constant (bold black line). In the absence of any FIH/ARD protein interaction, the non-CAD-hydroxylated form of HIFα decreases gradually with increasing oxygen (solid green line, $A_{tot} = 0$). In the presence of the FIH/AR-interaction by contrast, an oxygen threshold is introduced, and the drop in non-CAD-hydroxylated HIFα becomes very sharp (solid green line, $A_{tot} = 100$). Note that in the absence of PHD-activity, CAD-hydroxylation approaches completion at high oxygen levels (red dashed lines).

Figure S3

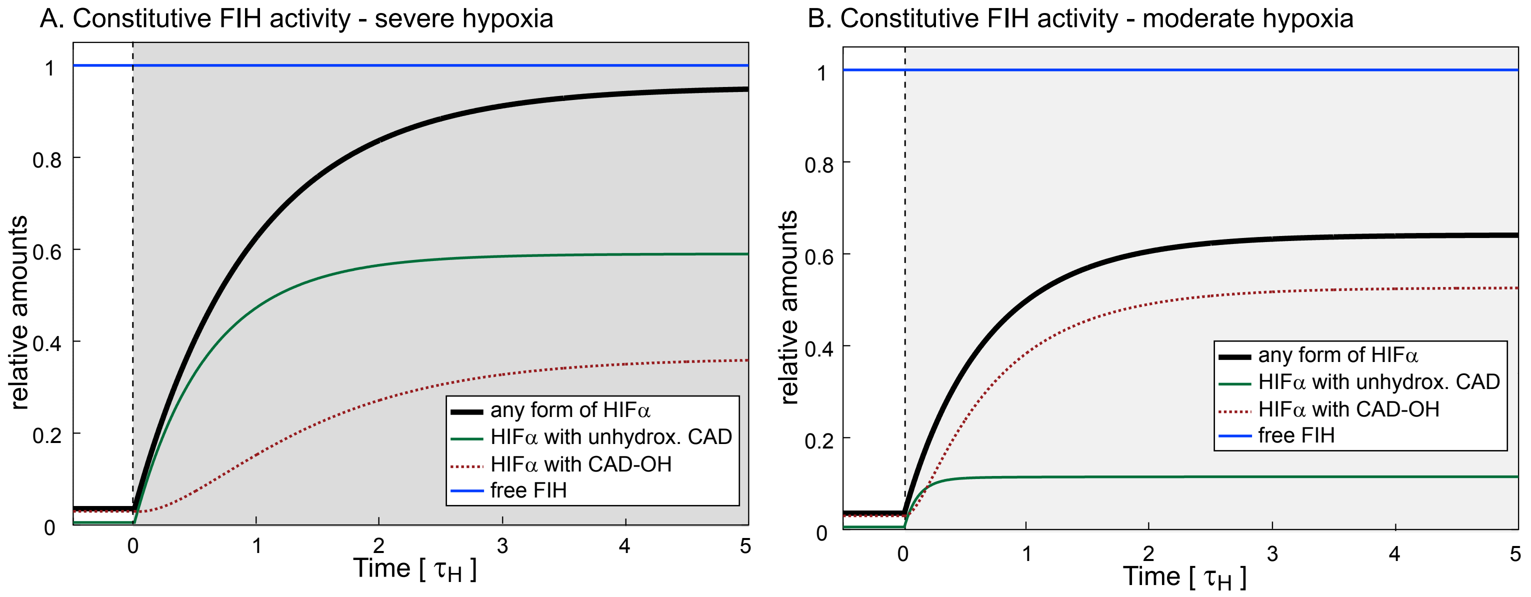

Figure S3. Full Model Simulation. Time course behaviour in response to hypoxia without an FIH/AR - interaction. Response to a step change from normoxia ($\tilde{O}_2 = 0.5$) to severe ($\tilde{O}_2 = 0.001$, A, dark grey area) or moderate hypoxia ($\tilde{O}_2 = 0.01$, B, light grey area) at $t = 0$. Parameters were $A_{tot} = 100$, $\gamma = 0.02$ and $\varepsilon = 5$. Time is given in units of the mean life time of of HIFα in the absence of oxygen.

Figure S4

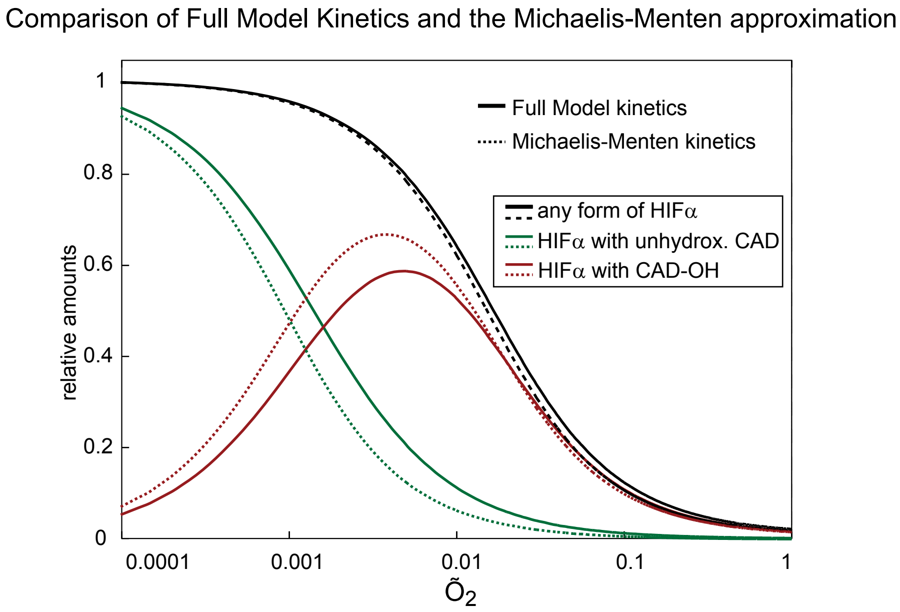

Figure S4. Comparing the Michaelis-Menten approximation with the kinetics used in the Full Model. HIFα-CAD-hydroxylation in the absence of ARD proteins is shown calculated by either Full Model kinetics (solid lines, the curves correspond to the case $A_{tot} = 0$ in Figure 5A, right hand panel) or Michaelis-Menten kinetics (dotted lines). The Full Model kinetics take into account that the concentration of free HIFα is decreased by binding to either FIH or PHD.
